# Investigating the Efficient Use of Word Embedding with Neural-Topic Models for Interpretable Topics from Short Texts

**DOI:** 10.3390/s22030852

**Published:** 2022-01-23

**Authors:** Riki Murakami, Basabi Chakraborty

**Affiliations:** 1 Graduate School of Software and Information Science, Iwate Prefectural University, 152-52 Sugo, Takizawa 020-0693, Iwate, Japan; g236r005@s.iwate-pu.ac.jp; 2 Faculty of Software and Information Science, Iwate Prefectural University, 152-52 Sugo, Takizawa 020-0693, Iwate, Japan

**Keywords:** short-text data, neural-topic model, pretrained word embedding, coherent topic, fine-tuning

## Abstract

With the rapid proliferation of social networking sites (SNS), automatic topic extraction from various text messages posted on SNS are becoming an important source of information for understanding current social trends or needs. Latent Dirichlet Allocation (LDA), a probabilistic generative model, is one of the popular topic models in the area of Natural Language Processing (NLP) and has been widely used in information retrieval, topic extraction, and document analysis. Unlike long texts from formal documents, messages on SNS are generally short. Traditional topic models such as LDA or pLSA (probabilistic latent semantic analysis) suffer performance degradation for short-text analysis due to a lack of word co-occurrence information in each short text. To cope with this problem, various techniques are evolving for interpretable topic modeling for short texts, pretrained word embedding with an external corpus combined with topic models is one of them. Due to recent developments of deep neural networks (DNN) and deep generative models, neural-topic models (NTM) are emerging to achieve flexibility and high performance in topic modeling. However, there are very few research works on neural-topic models with pretrained word embedding for generating high-quality topics from short texts. In this work, in addition to pretrained word embedding, a fine-tuning stage with an original corpus is proposed for training neural-topic models in order to generate semantically coherent, corpus-specific topics. An extensive study with eight neural-topic models has been completed to check the effectiveness of additional fine-tuning and pretrained word embedding in generating interpretable topics by simulation experiments with several benchmark datasets. The extracted topics are evaluated by different metrics of topic coherence and topic diversity. We have also studied the performance of the models in classification and clustering tasks. Our study concludes that though auxiliary word embedding with a large external corpus improves the topic coherency of short texts, an additional fine-tuning stage is needed for generating more corpus-specific topics from short-text data.

## 1. Introduction

Due to the rapid developments of computing and communication technologies and the widespread use of internet, people are gradually becoming accustomed to communicating through various online social platforms, such as microblogs, Twitter, webpages, Facebook, etc. These messages over web and social networking sites contain important information regarding current social situations and trends, people’s opinions on different products and services, advertisements, and announcements of government policies, etc. An efficient text-processing technique is needed to automatically analyze these huge amounts of messages for extracting information. In the area of traditional natural language processing, a topic-modeling algorithm is considered an effective technique for the semantic understanding of text documents. Conventional topic models, such as pLSA [1] or LDA [2] and their various variants, are considerably good at extracting latent semantic structures from a text corpus without prior annotations and are widely used in emerging topic detection, document classification, comment summarizing, or event tracking. In these models, documents are viewed as a mixture of topics, while each topic is viewed as a particular distribution over all the words. Statistical tools are used to determine the latent topic distribution of each document, while higher-order word co-occurrence patterns are used to characterize each topic [3]. The efficient capture of document-level word co-occurrence patterns leads to the success of topic modeling.

The messages posted on various social network sites are generally short compared to the length of relatively formal documents such as newspapers or scientific articles. The main characteristics of these short texts are: (1) a limited number of words in one document, (2) the use of new and informal words, (3) meanings and usages of words that may change greatly depending on the posting, (4) spam posts, and (5) the restricted length of posts, such as API restrictions on Twitter. The direct application of traditional topic models for short-text analysis results in poor performance due to lack of word co-occurrence information in each short text document, originating from the above characteristics of short texts [4]. Earlier research on topic-modeling for short texts with traditional topic models used external, large-scale datasets such as Wikipedia, or related long-text datasets for a better estimation of word co-occurrences across short texts [5,6]. However, these methods work well only when the external dataset closely matches the original short-text data.

To cope with the problems of short-text topic-modeling by traditional topic models, three main categories of algorithms are found in the literature [7]. A simple solution is to aggregate a number of short texts into a long pseudo-document before training a standard topic model to improve word co-occurrence information. In [8], the tweets of an individual user are aggregated in one document. In [9,10], a short text is viewed as sampled from unobserved, long pseudo-documents, and topics are inferred from them. However, the performance of these methods depends on efficient aggregation and data type. When short texts of different semantic contents are aggregated to a long document, non-semantic word co-occurrence information can produce incoherent topics. In the second category, each short text document is assumed to consist of only a single topic. Based on this assumption, Direchlet Multinomial Mixture (DMM) model-based topic-modeling methods have been developed for short texts in [11,12,13]. Although this simple assumption eliminates data-sparsity problems to some extent, they fail to capture multiple topic elements in a document, which makes the model prone to over-fitting. Moreover, “shortness” is subjective and data-dependent; a single-topic assumption might be too strong for some datasets. A poisson-based DMM model (PDMM) [14] considers a small number of topics associated with each short text instead of only one. The third category of algorithms consider global word co-occurrence patterns for inferring latent topics. According to the usage, two types of models are developed. In [15], global word co-occurrence is directly used, while in [16], a word co-occurrence network is first constructed using global word co-occurrence, and then latent topics are inferred from this network. In the present work, we explored methods of exploiting this category for further improvement in the development of algorithms for extracting interpretable topics from short texts.

Another limitation of the above models for short-text analysis is that the context or background information is not used, resulting in the generation of not-so-coherent topics. The statistical information of words in the text document cannot fully capture words that are semantically correlated but that rarely co-occur. Recent advances in word embedding [17] provides an effective way of learning semantic word relations from a large corpus, which can help to develop models for generating more interpretable and coherent topics. Word embedding uses one-hot representation of words with vocabulary length vectors of zeroes with a single one, and words that are similar in semantics are close in a lower-dimensional vector space. An embedded topic model (ETM), a combination of LDA and word embedding which enjoys both the advantages of topic model and word embedding, has been proposed in [18]. Traditional topic models with word embedding for documents are explored in several other research works, cited in [19]. In [20], word embedding is combined with LDA to accelerate the inference process, resulting in the enhanced interpretability of topics. For short texts, models incorporating word embedding into DMM are proposed in [21,22]. In [23,24], short texts are merged into long pseudo-documents using word embedding. Word embedding in conjunction with conventional topic models seems to be a better technique for generating coherent topics.

The increasing complexity of inference processes in conventional topic models on large-text data, along with the recent developments of deep neural networks, has led to the emergence of neural-topic models (NTM). These models combine the performance, efficacy, scalability, and ease of leveraging parallel computing facilities, such as GPU, to probabilistic topic-modeling [25]. Neural-topic models are considered to be computationally simpler and easier for implementation, compared to traditional LDA models, and are increasingly used in various natural language processing tasks in which conventional topic models are difficult to use. A systematic study on the performances of several neural-topic models has been reported in [26]. Although various neural-topic models have been proposed, and although the reported experimental results on topic generation seem to be better than conventional topic models for long and formal texts, little research has been conducted on neural-topic models for effective analysis of short texts [27]. Most of the research works of topic modeling on short texts are based on extensions of Bayesian probabilistic topic models (BPTM) such as LDA.

The objective of the present research is to explore computationally easy and efficient techniques for improving the interpretability of generated topics from real-world short texts using neural-topic models. However, learning context information is the most challenging issue of topic-modeling for short texts, and incorporating pretrained word embedding into a topic model seems to be one of the most efficient ways of explicitly enriching the content information. Neural-topic models with pretrained word embedding for short-text analysis has not been extensively explored yet, compared to its long-text counterparts. In [28], we presented our preliminary analysis of short-text data (benchmark and real-world) with neural-topic models using pretrained word embedding. We found that although pretrained word embedding enhances the topic coherence of short texts that are similar to long and formal texts, the generated topics were often comprised of words having common meanings (which are found in the large external corpus used for pretraining) instead of the particular short-text-specific semantics of the word, which is especially important for real-world datasets. In other words, the learning of topic centroid vectors is influenced by pretraining the text corpus and fails to discover the important words of the particular short text. Our proposal is that this gap can be filled by adding a fine-tuning stage to the training of the topic model with the particular short-text corpus to be analysed. In this work, we have completed an extensive study to investigate the performance of recent neural-topic models with and without word embedding, and also with the proposed fine-tuning stage, for generating interpretable topics from short texts in terms of a number of performance metrics by simulation experiments on several datasets. We have also studied the performance of the NTM with pretrained word embedding added, with a fine-tuning stage for classification and clustering tasks. As a result of our experiments, we can confirm that the addition of a fine-tuning stage indeed enhances the topic quality of short texts in general, and generates topics with corpus-specific semantics.

In summary, our contributions in this paper are as follows:

- A proposal for fine-tuning with the original short-text corpus, along with the pretrained word embedding with the large external corpus, for generating more interpretable and coherent corpus-specific topics from short texts;
- An extensive evaluation of the performance of several neural-topic models, with and without pretrained word embedding and with an added fine-tuning stage, in terms of topic quality and measured by several metrics of topic coherence and topic diversity;
- A performance evaluation of the proposed fine-tuned neural-topic models for classification and clustering tasks.

In the next section, neural-topic models are introduced in brief, followed by a short description of related works on neural-topic models (NTM), especially NTMs for short texts. The following sections contain our proposal, followed by simulation experiments and results. The final section presents the conclusion.

## 2. Neural-Topic Models and Related Works

The most popular neural-topic models (NTMs) are based on a variational autoencoder (VAE) [29], a deep generative model, and amortised variational inferences (AVI) [30]. The basic framework of VAE-based NTMs is described in the next section, in which generative and inference processes are modeled by a neural network-based decoder and encoder, respectively. Compared to the traditional Bayesian probabilistic topic models (BPTM), inference in neural-topic models is computationally simpler, their implementation is easier due to many existing deep learning frameworks, and NTMs are easy to be integrated with pretrained word embeddings for prior-knowledge acquisition. Several categories of VAE-based NTMs have been proposed. To name a few, there is the Neural Variational Document Model (NVDM) [31], Neural Variational Latent Dirichlet Allocation (NVLDA) [32], the Dirichlet Variational Autoencoder topic model (DVAE) [33], the Dirichlet Variational Autoencoder (DirVAE) [34], the Gaussian Softmax Model (GSM) [35], and iTM-VAE [36]. This list is not exhaustive and is still growing.

In addition to VAE-based NTMs, there are a few other frameworks for NTMs. In [37], an autoregressive NTM, named DocNade, has been proposed. Consequently, some extensions of DocNADE are found in the literature. Recently, some attempts have been made to use a GAN (Generative Adversarial Network) framework for topic-modeling [38,39]. Instead of considering a document as a sequence or a bag of words, a graph representation of a corpus of documents can be considered. In [40], a bipartite graph, with documents and words as two separate partitions and connected by word occurrences in documents as the weights, is used. Ref. [41] uses the framework of Wasserstein auto-encoders (WAEs), which minimizes the Wasserstein distance between reconstructed documents from the decoder and the real documents, similar to a VAE-based NTM. In [42], a NTM based on optimal transport that directly minimizes the optimal transport distance between the topic distribution learned by an encoder and the word distribution of a document has been introduced.

### Neural-Topic Models for Short-Text Analysis

In order to generate coherent, meaningful, and interpretable topics from short texts by incorporating semantic and contextual information, a few researchers used NTMs in lieu of conventional topic models. In [43,44], a combination of NTM and either a recurrent neural network (RNN) or a memory network has been used, in which topics learned by the NTM are utilized for classification by a RNN or a memory network. In both works, the NTM shows better performance than conventional topic models in terms of topic coherence and a classification task. To enhance the discreteness of multiple topic distributions in a short text, in [27], the authors used Archimedean copulas. In [45], the authors introduced a new NTM with a topic-distribution quantization approach, producing peakier distributions, and also proposed a negative sampling decode, learning to minimize repetitive topics. As a result, the proposed model outperforms conventional topic models. In [46], the authors aggregated short texts into long documents and incorporated document embedding to provide word co-occurrence information. In [47], a variational autoencoder topic model (VAETM) and a supervised version (SVAETM) of it have been proposed by combining embedded representations of words and entities by employing an external corpus. To enhance contextual information, the authors in [48] proposed a graph neural network as the encoder of NTM, which accepts a bi-term graph of the words as inputs and produces the topic distribution of the corpus as the output. Ref. [49] proposed a context-reinforced neural-topic model with the assumption of a few salient topics for each short text, informing the word distributions of the topics using pretrained word embedding.

## 3. Proposal for Fine-Tuning of Neural-Topic Models for Short-Text Analysis

From the analysis of present research works on neural-topic models on short-text analysis, it seems that incorporating auxiliary information from an external corpus is one of the most popular and effective techniques for dealing with sparsity in short texts. As mentioned in the introduction, in our previous work [28], we found that although pretrained word embedding with a large external corpus helps with generating coherent topics from a short-text corpus, the generated topics lack the semantics expressed by the corpus-specific meaning of words. If the domain of the short-text corpus and the external corpus vary too much, the topic semantics become poor. This fact is also noted by other researchers [25].

**Figure 1:**
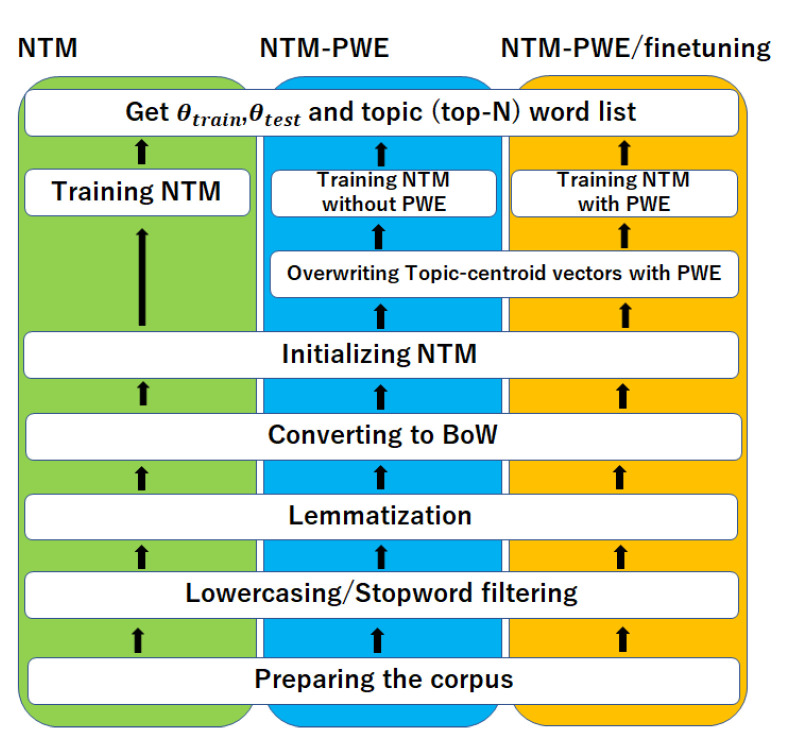
Proposed Study.

In this work, we propose an additional fine-tuning stage, using the original short-text corpus, along with the pretrained word embedding and a large external corpus. For pretrained word embedding, we decided to use GloVe [50] after some preliminary experiments with two other techniques, namely, Word2Vec and Fast Text, as GloVe provided consistent results. Here, we have completed an extensive comparative study to evaluate the effect of pretrained embedding with our proposed additional fine-tuning stage using several short-text corpora and neural-topic models. The proposed study setting is represented in Figure 1.

Here, pretrained word embedding is denoted as PWE. We have performed three sets of experiments for topic extraction, using only neural-topic models (NTM), neural-topic models with pretrained word embedding (NTM-PWE), and neural-topic models with pretrained embedding and the proposed fine-tuning step (NTM-PWE/fine-tuning). In all the cases, the data corpus is first pre-processed, and in NTM-PWE, word embedding vectors are replaced by PWE after the model parameters are initialized; the weights of PWE are not updated during the training step. In our proposed PWE/fine-tuning or simple fine-tuning (as mentioned in the text), the weights are gradually updated in the training step after replacing the word embedding vectors, as in PWE. In this case, it is possible to update the parameters at the same learning rate that is set to update the entire model, but experiments have shown that updating the PWE values at a large learning rate can easily over-fit the training data. Therefore, in the simulation experiments, we have set the learning rate of the word embedding vectors to a smaller value than the learning rate of the entire model.

We have used popular VAE-based neural-topic models with a few similar WAE (Wasserstein autoencoder)-based models, and ten popular benchmark datasets, for our simulation experiments. The performance of each neural-topic model with no word embedding, pretrained word embedding, and additional fine-tuning has been evaluated by the generated topic quality using different evaluation metrics of topic coherence and topic diversity. The neural-topic models, datasets, and evaluation metrics used in this study are described below.

### 3.1. Neural-Topic Models for Evaluation

In this section, the neural-topic models used in this study are described briefly. Table 1 describes the meaning of the notations used for description of the models.

**Figure 2:**
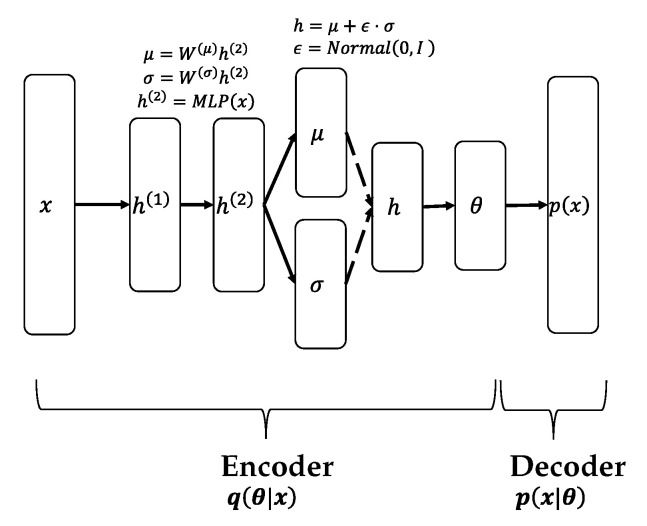
VAE-based model.

**Figure 3:**
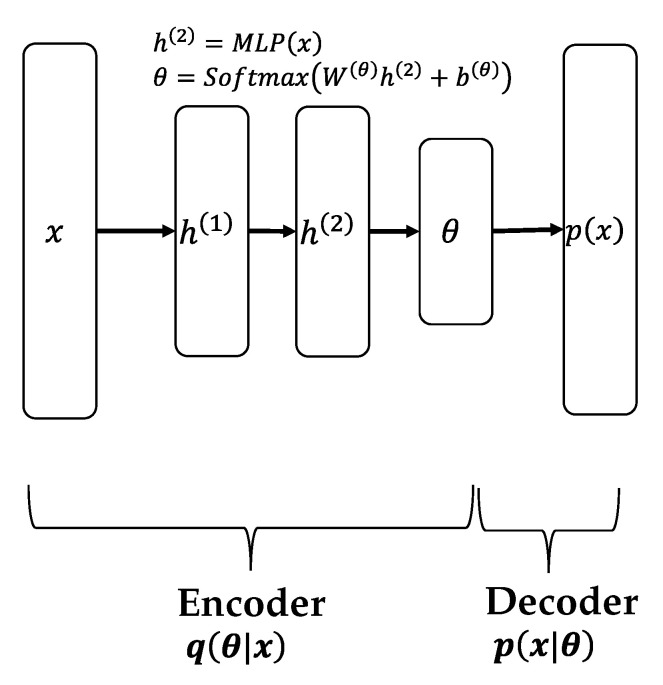
WAE-based model.

Figure 2 and Figure 3 describe the generalized architecture of the Variational autoencoder (VAE)- and Wasserstein autoencoder (WAE)-based neural-topic models, respectively. In both the models, the part of the network that generates θ is known as the encoder, which maps the input bag-of-words (BoW) to a latent document–topic vector, and the part that receives θ and outputs p(x) is called the decoder, which maps the document–topic vector to a discrete distribution over the words in the vocabulary. They are called autoencoders because the decoder aims to reconstruct the word distribution of the input. In VAE, *h* is sampled by Gaussian distribution, and θ is created by performing some transformation on it. WAE, on the other hand, uses the Softmax function directly to create θ, so no sampling is required. Evidence lower bound (ELBO), the objective function of VAE, is defined below [29]:

(1) $$\begin{array}{cc} L_{d}=\; & E_{q(\theta |d)}\left[\sum_{n=1}^{N_{d}}\log\sum_{z_{n}}\left[p\left(w_{n}|\beta_{z_{n}}\right)p\left(z_{n}|\theta \right)\right]\right]\\ \; & -D_{KL}\left[q\left(\theta |x_{d}\right)∥p\left(\theta |\mu_{0},\sigma_{0}^{2}\right)\right] \end{array}$$

It is empirically known that maximizing this ELBO alone will result in smaller (worse) topic diversity. In order to solve this problem, some NTMs use a regularization term to increase the topic diversity [51]:

(2) $$\begin{array}{c} a\left(\alpha_{i},\alpha_{j}\right)=\arccos\left(\frac{\left|\alpha_{i}\cdot \alpha_{j}\right|}{∥\alpha_{i}∥\cdot ∥\alpha_{j}∥}\right)\\ \zeta =\frac{1}{K^{2}}\sum_{i}^{K}\sum_{j}^{K}a\left(\alpha_{i},\alpha_{j}\right)\\ \nu =\frac{1}{K^{2}}\sum_{i}^{K}\sum_{j}^{K}{\left(a\left(\alpha_{i},\alpha_{j}\right)-\zeta \right)}^{2}\\ J=L+\lambda (\zeta -\nu ) \end{array}$$

where λ is a hyper-parameter that manipulates the influence of the regularization term; 10 was adopted here. This value was determined empirically. The VAE-based models in this paper use this regularization term.

The particular NTMs used in our study are mentioned in the next subsections.

#### 3.1.1. Neural Variational Document Model (NVDM)

NVDM [31] is, to our knowledge, the first VAE-based document model proposed with the encoder implemented by a multilayer perceptron. This model uses the sample *h* from the Gaussian distribution as an input for the decoder, and variational inference is based on minimizing KL divergence. While most of the NTMs proposed after this one transform *h* to treat θ as a topic proportion vector, NVDM is a general VAE.

#### 3.1.2. Neural Variational Latent Dirichlet Allocation (NVLDA)

NVLDA [32], another variant of NVDM, is a model that uses Neural Variational Inference to reproduce LDA. Here, the Softmax function is used to convert z to θ. The probability distribution that maps samples from a Gaussian distribution to the Softmax basis is called the Logisitic–Normal distribution, which is used as a surrogate for the Dirichlet distribution. Additionally, the decoder is p(x)=softmax(β)·θ. Unlike the NVDM, where both the topic proportions and the topic–word distribution are in the form of probability distributions, this model is a topic model. Logisitic–Normal distribution is defined as follows:

(3) $$\begin{array}{c} h∼Normal(\mu ,\sigma^{2}) \end{array}$$

(4) $$\begin{array}{c} \theta =softmax(h) \end{array}$$

#### 3.1.3. Product-of-Experts Latent Dirichlet Allocation (ProdLDA)

ProdLDA [32] is an extension of NVLDA in which the decoder is designed by following the product of the expert model, and the topics-word distribution are not normalized.

#### 3.1.4. Gaussian Softmax Model (GSM)

GSM [35] converts *h* to θ using Gaussian Softmax, as defined below:

(5) $$\begin{array}{c} h∼Normal(\mu ,\sigma^{2}) \end{array}$$

(6) $$\begin{array}{c} \theta =\mathrm{softmax}(W_{1}^{T}h) \end{array}$$

where $W_{1}\in R^{K\times K}$, a linear transformation, is the trainable parameters used as the connection weights.

#### 3.1.5. Gaussian Stick-Breaking Model (GSB)

GSB [35] converts *h* to θ by Gaussian Stick-Breaking construction, which is defined as follows:

(7) $$\begin{array}{c} h∼Normal(\mu ,\sigma^{2}) \end{array}$$

(8) $$\begin{array}{c} \eta =sigmoid(W_{2}^{T}h) \end{array}$$

(9) $$\begin{array}{c} \theta =f_{SB}\left(\eta \right) \end{array}$$

where $W_{2}\in R^{K\times K-1}$ is the trainable parameters used as connection weights, and the stick-breaking function $f_{SB}$ is described by Algorithm 1:
**Algorithm 1** Stick-Breaking Process ($f_{SB}$)**Input:** Return value from sigmoid function η$\eta \in R_{+}^{K}$, where $\forall \eta_{k}\in \left[0,1\right]$**Output:** Topic proportion vector θ$\theta \in R_{+}^{K}$, where $\sum_{k}\left(\theta_{k}\right)=1$1:Assign $\eta_{1}$ to the first element of the topic proportion vector $\theta_{1}$.$\theta_{1}=\eta_{1}$2:**for**k=2,…K−1**do**$$\theta_{k}=\eta_{k}\prod_{i=1}^{k-1}\left(1-\eta_{i}\right)$$3:**end for**4:$$\theta_{K}=\prod_{i=1}^{K-1}\left(1-\eta_{i}\right)$$

#### 3.1.6. Recurrent Stick-Breaking Model (RSB)

RSB [35] converts *h* to θ by recurrent Stick-Breaking construction, as defined below. Here, the stick-breaking construction is considered as a sequential draw from a recurrent neural network (RNN).

(10) $$\begin{array}{c} h∼Normal(\mu ,\sigma^{2}) \end{array}$$

(11) $$\begin{array}{c} \eta =f_{\mathrm{RNN}}\left(h\right) \end{array}$$

(12) $$\begin{array}{c} \theta =f_{\mathrm{SB}}\left(\eta \right) \end{array}$$

where $f_{\mathrm{RNN}}$ is decomposed as:

(13) $$\begin{array}{c} h_{k}={\mathrm{RNN}}_{\mathrm{SB}}\left(h_{k-1}\right) \end{array}$$

(14) $$\begin{array}{c} \eta_{k}=\mathrm{sigmoid}\left(h_{k-1}^{T}z\right) \end{array}$$

and $f_{SB}\left(\eta \right)$ is the same as in GSB.

#### 3.1.7. Wasserstein Latent Dirichlet Allocation (WLDA)

WLDA [41] is a topic model based on a Wasserstein autoencoder (WAE). Though various probability distributions can be used for the prior distribution of θ, in this paper, we use the Dirichlet distribution, which we believe is the most basic. In WAE, two training methods are available, GAN (Generative Adversarial Network)-based training and MMD (Maximum Mean Discrepancy)-based training, but in WLDA, MMD is used because of the ease of convergence of training loss. In VAE, the loss function is composed of the KL Divergence used as the regularization term for θ and the reconstruction error, while in WLDA, MMD is used as the regularization term.

If $P_{\Theta }$ is a θ’s prior distribution, and $Q_{\Theta }$ is a fake samples‘s prior distribution, maximum mean discrepancy (MMD) is defined as:

(15) $$\begin{array}{c} {\mathrm{MMD}}_{k}\left(Q_{\Theta },P_{\Theta }\right)={∥\int_{\Theta }k\left(\theta ,\cdot \right)dP_{\Theta }\left(\theta \right)-\int_{\Theta }k\left(\theta ,\cdot \right)dQ_{\Theta }\left(\theta \right)∥}_{H_{k}} \end{array}$$

where H means the reproducing kernel Hilbert space (RKHS) of real-valued functions mapping Θ to R, and *k* is the kernel function.

(16) $$\begin{array}{c} k\left(\theta ,\theta^{\prime }\right)=\exp\left(-\arccos^{2}\left(\sum_{k=1}^{K}\sqrt{\theta_{k}\theta_{k}^{\prime }}\right)\right) \end{array}$$

#### 3.1.8. Neural Sinkhorn Topic Model (NSTM)

NSTM [52] is trained using optimal transport [42], as in WLDA. Since we assume that θ encodes *x* into a low-dimensional latent space while preserving sufficient information about *x*, the optimal transport distance between θ and *x* is calculated by the Sinkhorn Algorithm. The sum of this optimal transport distance and the negative log likelihood is used as the loss function.

### 3.2. Datasets

Table 2 presents the details of the benchmark datasets used in this work. The first column represents the name of the dataset, followed by the number of documents (|D|), vocabulary size (|V|), the total number of tokens (∑X), average document length (ave dL), maximum document length (max dL), sparsity, number of classes (C,) and the source of the data in the respective columns. In the source column, 1 and 2 represent OCCITS and STTM, respectively.

1. OCTIS: https://aclanthology.org/2021.eacl-demos.31/ (accessed on 14 January 2022).

2. STTM: https://arxiv.org/pdf/1701.00185.pdf (accessed on 14 January 2022).

The first two datasets fall in the category of long documents, and the other eight datasets can be considered as the short-text corpus, as the average document length is quite short compared to the long documents.

The datasets shown in the table are pre-processed. HTML tags and other symbols have been removed from each dataset, and all words have been lowercased. Then, the stop-words were removed and lemmatized. From these datasets, 80% of the total documents was used as the training data and the rest as the test data. These pre-processed corpora are then converted into a BoW (Bag-of-Words), which basically has word frequency as an element, to be used as input data for the NTM. However, for the NSTM, the vector corresponding to each document in the BoW is divided by the total value of the vectors, as in the original paper.

### 3.3. Evaluation of Topic Quality

It is quite challenging to evaluate the performance of topic models, including NTMs, according to the quality of the generated topics. Topics generated by topic models can be considered as soft clusters of words. Under the constraints of the topic model, this is a probability distribution that collects the probability of word generation for each topic; the same is true for NTM, but this may not be in the form of a probability distribution for document models that impose even weaker constraints than the topic model. Either way, a topic here is a topic–word distribution, and each distribution has as many dimensions as the number of lexemes that occur in the corpus. It is very difficult to understand the goodness of a topic by directly comparing them with human topics. Therefore, in practice, analysts check a list of N words characteristic of a topic based on the values of the word distributions. In most cases, the list of the top-N words in terms of the large probability values in the word distribution is used.

Various metrics have been proposed to evaluate the quality of the top-N words with two main directions. One is to check whether the meaning of words belonging to the top-N words are consistent with each other, defined as topic coherence (TC). The other is to measure the diversity of the top-N words of each pair of topics, defined as topic diversity (TD) or topic uniqueness. Topics with high TC may have low TD. In this case, the top-N words of most topics will be nearly the same, which is not desirable. So, to evaluate the quality of a topic for human-like interpretability, it should have high TC as well as high TD.

#### 3.3.1. Topic Coherence (TC)

For computing TC, general coherence between two sets of words are estimated based on word co-occurrence counts in a reference corpus [53]. The choices are (1) the training corpus for topic modeling; (2) a large external corpus (e.g., Wikipedia); (3) word embedding vectors trained on a large external corpus (e.g., Wikipedia). The scores may differ according to different computations. Choice 1 is easy, but the results are affected by the size of the training corpus. Choices 2 and 3 are more popular, although choice 2 is computationally costly. However, if the domain gap of the training corpus and the external corpus is high, the evaluation is not proper. In this work, we have used the following metrics for computation of topic coherence:

- Normalized Point-Wise Mutual Information (NPMI) [54]: NPMI is a measure of the semantic coherence of a group of words. It is considered to have the largest correlations with human ratings, and is defined by the following equation:
  (17) $$NPMI\left(w\right)=\frac{1}{N(N-1)}\sum_{j=2}^{N}\sum_{i=1}^{j-1}\frac{log\frac{P(w_{i},w_{j})}{P\left(w_{i}\right)P\left(w_{j}\right)}}{-logP(w_{i},w_{j})}$$
  where *w* is the list of the top-N words for a topic. N is usually set to 10. For K topics, averages of NPMI over all topics are used for evaluation;
- Word Embeddings Topic Coherence (WETC) [55]: WETC represents word embedding-based topic coherence, and pair-wise WETC for a particular topic is defined as:
  (18) $${\mathrm{WETC}}_{PW}\left(E^{\left(k\right)}\right)=\frac{1}{N(N-1)}\sum_{j=2}^{N}\sum_{i=1}^{j-1}\langle E_{i,:}^{\left(k\right)},E_{j,:}^{\left(k\right)}\rangle$$
  where 〈.,.〉 denotes the inner product. For the calculation of the WETC score, pretrained weights of GloVe [50] have been used, and $E^{\left(k\right)}$ is the word embedding vector sequence of GloVe corresponding to the top-N words for topic *k*; $E_{i}^{\left(k\right)}$ means and all vectors are normalized as follows: $\left|\right|E_{i,:}^{\left(k\right)}\left|\right|=1$, *N* is taken as 10.
  ${\mathrm{WETC}}_{c}$ (centroid WETC) is defined as follows:
  (19) $$\begin{array}{cc} {\mathrm{WETC}}_{c}\left(E^{\left(k\right)}\right) & =\frac{1}{N}\sum_{n=1}^{N}E^{\left(k\right)}t\\ t & =\frac{\alpha_{:,k}}{\left|\right|\alpha_{:,k}\left|\right|} \end{array}$$

#### 3.3.2. Topic Diversity

Topic diversity is defined here as the percentage of unique words in the top 25 words of all topics, according to [18]. A diversity close to 0 represents a redundant topic, and those close to 1 indicate more varied topics. Here, we have also used two other metrics, inverted rank-biased overlap (InvertedRBO) [56] and mean squared cosine deviation among topics (MSCD) [57], as a measure of diversity of the generated topics. InvertedRBO is a measure of disjointedness between topics weighted on word rankings, based on the top-N words. The higher these metrics are, the better. MSCD is the cosine similarity of the word distribution of each topic, so it should be lower for better topics. In general, NTM training updates parameters to maximize ELBO, but such a naive implementation can easily lead to poor TD. However, in this case, since we use the topic centroid vectors as trainable parameters, we regularize parameters of NTM to increase the angle formed by each topic centroid vector in order to increase the TD.

## 4. Simulation Experiments and Results

The simulation experiments have been performed with several benchmark datasets, and the performance of the topic models are evaluated by topic coherence and topic diversity measures.

### 4.1. Experimental Configuration

For the purpose of comparison and evaluation, the experimental setting should be similar for all the neural-topic models and all the datasets. At the beginning, we completed some trial experiments, and determined that the optimum topic size parameter should be set at K=50, based on topic coherence and perplexity, so that there are a sufficient number of topics without becoming very large, considering the length of short text. This value is also in accordance with the value used for related experiments in similar research works. The number of dimensions of the word embeddings was fixed at L=300. This is in accordance with the GloVe’s Common Crawl-based trained word embedding vectors, publicly available at https://nlp.stanford.edu/projects/glove/ (accessed on 14 January 2022), which cover largest number of vocabularies.

The other experimental parameters are set as follows: number of units of the encoder’s hidden layers: $H^{\left(1\right)}=500,H^{\left(2\right)}=500$; Dropout rate: $p_{\mathrm{dropout}}=0.2$; Minibatch size: 256; Max epochs: 200; Learning rate for the encoder network: 0.005; Learning rate for the decoder network: 0.001. We employ Adam as the optimizer and Softplus as the activation function of the encoder networks.

On WLDA, we employ Dirichlet as the prior distribution for topic proportion-generation, using MMD for this model’s training. On NSTM, Sinkhorn algorithm’s max number of updates is 2000, and the threshold value for updating termination condition is 0.05, constant value $\alpha_{\mathrm{Sinkhorn}}=20$.

### 4.2. Results for Topic Coherence

Table 3, Table 4, Table 5, Table 6, Table 7, Table 8, Table 9, Table 10, Table 11 and Table 12 represent the detail results of different topic coherence metrics (NPMI and WETC) for different neural models and different datasets, respectively. Values in bold faces indicate the best results. We have used two versions of GloVe, differing in terms of the size of the corpus.

For many datasets, NVLDA-PWE/fine-tuning has the highest TC. One of the challenges of using PWE without fine-tuning is that the high domain gap between the PWE training corpus and the corpus for topic modeling has a negative impact. In many cases, our proposal produces better results, but not for all the datasets or for all the models. The dataset “GoogleNews” often has the best TC with PWE, and does not show better performance with additional fine-tuning. This is probably because this corpus has a similar domain as the training data for PWE. In a few datasets, the best performance is noticed when no pretraining word embedding is used. It is verified that for those datasets, the original corpus contains sufficient word co-occurrence information.

However, we noted that the TC value changes significantly depending on the type of word embedding. This result suggests that the quality of the word embeddings may have a significant impact on the training of the topic model. In particular, whether or not the unique words in the training corpus are included in the unique words in the PWE has a significant impact. If the coverage of this word dictionary is large, the PWE can be used for evaluation, but if there are many missing words, the reliability of the evaluation value will be greatly compromised.

**Figure 4:**
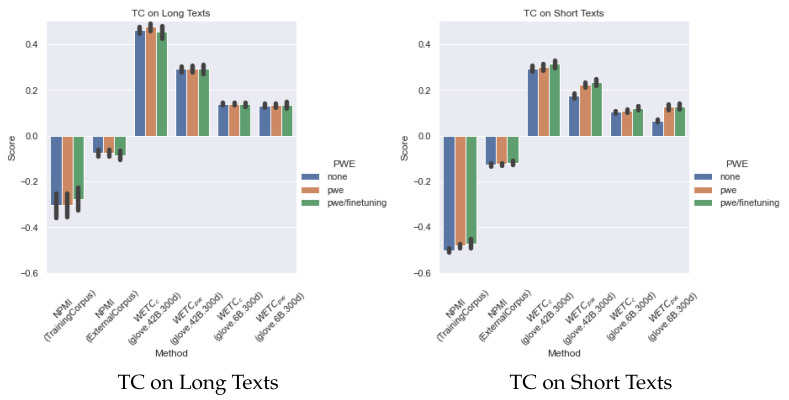
Summary of TC results.

Figure 4 presents the summary of topic coherence over all the neural-topic models for the long-text corpus (2 datasets) and the short-text corpus (8 datasets), which shows the overall trend. In the case of long texts, the scores of the PWE/fine-tuning metrics for TC are either a little worse or the same as the others. One of the reasons for this is that the long-text corpus used in this study is composed of relatively formal documents, which is close to the domain of PWE. In contrast, the short-text corpus shows better performance in all metrics. The overall trend is none < PWE < PWE/finetuning.

### 4.3. Results for Topic Diversity

Table 13, Table 14, Table 15, Table 16, Table 17, Table 18, Table 19, Table 20, Table 21 and Table 22 represent the detailed results of different metrics (TopicDiversity, Inverted RBO, and MSCD) expressing a diversity of topics for different neural-topic models and different datasets, respectively. Values in bold indicate the best results. InvertedRBO focuses on the weight of the top-N words. It shows the highest values in almost all of the cases, from which it can be inferred that we were able to construct the topics with high diversity. This result shows that it was useful to add a regularization term that maximizes the distance between topic-centroid vectors, resulting in highly diverse topics.

Furthermore, WLDA and NSTM show similar results without this regularization term, indicating that these models are able to learn without compromising topic diversity in their raw form. To check if this regularization term is working well, we have added TopicCentroidDistance (TCD) in the tables. The larger this metric is, the better, but the values are almost the same for all cases. This metric was evaluated based on two PWEs, and since the values varied, we can infer that the quality of the embedding has a significant impact on the evaluation of the topic model.

Although the results of TopicDiversity varied greatly depending on model and dataset, when checked individually, the scores were sufficiently better in many cases. However, as in the case of Biomedical’s NVLDA-pwe/fine-tuning results, there were cases where the TC showed good scores but the TD showed bad scores. In this respect, InvertedRBO also shows a good score, but MSCD, which is an evaluation using the entire topic–word distribution, shows a relatively large value (i.e., a bad score), indicating that the topics are relatively tangled. Metrics such as TopicDiversity and InvertedRBO, which are based on the top-N words, are useful for evaluating topic diversity, but it is also important to evaluate the entire topic–word distribution.

**Figure 5:**
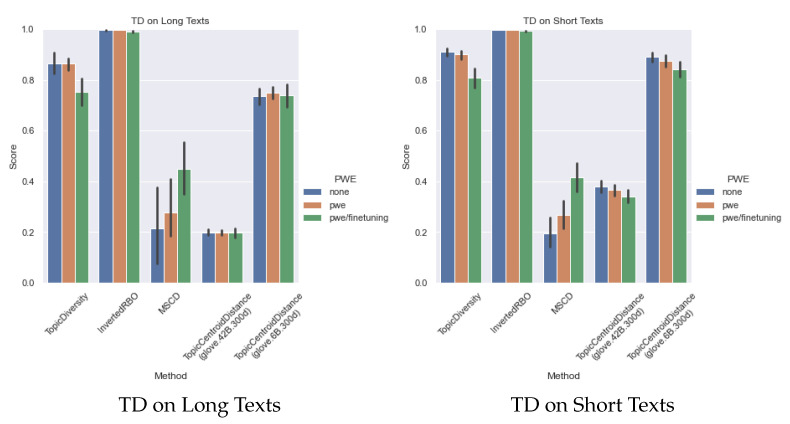
Summary of TD Results.

Figure 5 presents the summary of topic diversity results over all the neural-topic models for the long-text corpus (2 datasets) and the short-text corpus (8 datasets), which shows the overall trend. Among the metrics related to TD, the InvertedRBO score is almost the highest in all cases. This indicates that there is sufficient diversity in all conditions. However, for the other scores, the performance is slightly worse for PWE and PWE/fine-tuning.

### 4.4. Classification and Clustering Performance

**Figure 6:**
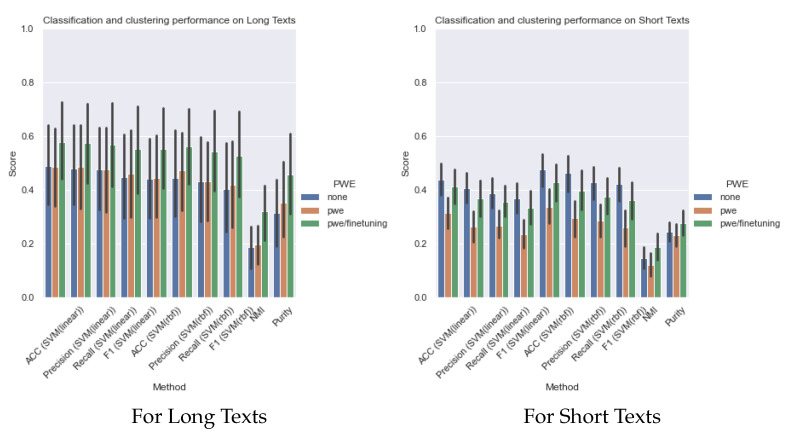
Classification and clustering performance.

Table 23, Table 24, Table 25, Table 26, Table 27, Table 28, Table 29, Table 30, Table 31 and Table 32 represent the classification and clustering performance of all models and all datasets, respectively. Values in the bold face represent best results. For the TrecTweet dataset, the classification results could not be obtained, possibly due to some technical problem. Figure 6 presents the average classification and clustering performance of the models over long- and short-text datasets. Classification has been performed by a SVM (Support Vector Machine) with linear and rbf kernels. Classification accuracy, precision, recall, and F1 scores have been used for performance assessment and for supervised classification, and NMI (Normalized Mutual Information) and Purity have been used for unsupervised classification.

For classification, VAE-based models, such as NVDM and GSM, exhibit good performance, while WAE-based models, such as WLDA and NSTM, show relatively poor performance. NSTM shows good performance in TC and TD, especially in TD, without adding any regularization term. However, the application to downstream tasks using WAE variants remains a challenge. Considering the overall trend, for long texts, PWE with fine-tuning improves all the scores, but for short texts, the performance is the best for the cases without embedding. Although, after fine-tuning, the scores got better than those obtained with pretrained embedding only.

For clustering results, the large NMI and Purity scores for all models and all datasets for both long and short texts indicate that there is a concentration of documents with the same label around the topic-centroid vector, which proves that the proposal of PWE/fine-tuning improves topic cohesion. Therefore, we can see that our proposal of PWE/fine-tuning contributes to narrowing the domain gap between the training corpus and PWE.

## 5. Conclusions

Short-text data are now becoming ubiquitous in the real world through various social networking sites. The importance of analysing these short messages is also growing day by day. Unlike long texts or documents, short texts suffer from a lack of word co-occurrence information due to their restricted lengths, posing a difficulty in generating coherent and interpretable topics with popular topic-model techniques.

The use of pretrained word embedding in neural-topic models is a good choice to easily increase the generated topic quality as measured by topic coherence and topic diversity. This is effective for both long and short texts, and reduces the number of trainable parameters, thus shortening the training step time. However, to achieve better topic coherence, especially in short texts, or to make the top-N words of a topic more relevant to the real semantic contents of the training corpus, the additional fine-tuning stage proposed in this work is indeed necessary. The extensive study in this work with several neural-topic models and benchmark datasets justifies our proposal.

However, the use of pretrained word embedding (PWE) has its inherent limitations, which may affect the quality of the extracted topics from short texts. The short-text corpus to be analyzed may contain words that are not included in the vocabulary covered by the corpus used for pretrained word embedding. In this case, NTM-PWE uses a vector initialized with zero. As the vocabulary coverage increases, the performance is likely to deteriorate. Moreover, in the case of NTM-PWE/fine-tuning, there is a possibility that the number of parameter updates will increase until the loss function converges, resulting in an increase in training time. If the time difference between the corpus used for PWE training and the corpus to be analyzed is too large, the meanings of words may change with time, which may have a negative impact on the production of interpretable topics.

It is also seen that the improvement in topic quality after introducing a fine-tuning stage is not the same for all the datasets and all the models. It is difficult to define the correlation between the structure of neural-topic models and the inherent characteristics of the datasets, which poses a challenge to our study. In this work, we limited our study to benchmark datasets available on the internet. Currently, we are collecting data for the evaluation of our proposal with real-world datasets.

By incorporating the additional training with the original training corpus, along with pretrained word embedding with the external corpus, we can improve the purity and NMI of the topics evaluated using the class labels of the documents. Thus, we can construct topics that are more suitable for the training corpus. This method can also be expected to improve the performance of downstream tasks, such as classifications for long texts. Even for short texts, the performance of the downstream tasks is better than when using pretrained word embedding without fine-tuning.

## Figures and Tables

**Table 1:** Table of notations used.

Indices:	
*K*	Number of topics k∈{1,…,K}
*L*	Word embedding vectors dimension l∈{1,…,L}
*C*	Number of classes c∈{1,…,C}
**Decision Variables:**	
*D*	Set of documents
*V*	Set of lexicons, vocabularies
*X*	BoW matrix of all documents, $X\in R_{+}^{\left|V|\times |D\right|}$
$x_{d}$	*d*’s BoW representation vector, $x_{d}\in R_{+}^{\left|V\right|}$
*N*	Number of words that occurred in document *d*
$w_{n}$	*n*-th word
**Random Variables:**	
$h^{\left(i\right)}$	*i*-th hidden layer’s outputs
*h*	Gaussian random variables, $h\in R^{K}$
$z_{n}$	latent topic for the *n*-th word
θ	Topic proportion vector, $\theta \in R_{+}^{K}$
β	Topic–word distribution $\beta \in R^{|V|\times K}$
α	Topic centroid vectors $\alpha \in R^{L\times K}$
ρ	Word embedding vectors $\rho \in R^{L\times |V|}$

**Table 2:** Details of Data set.

Name	|D|	|V|	∑X	ave dL	max dL	Sparsity	*C*	Source
BBC_news	2225	2949	267,259	120.12	1176	0.027811	5	1
20NewsGroup	16,309	1612	783,151	48.02	3033	0.018855	20	1
SearchSnippets	12,295	4720	177,338	14.42	37	0.002167	8	2
TrecTweet	2472	5098	21,148	8.56	20	0.001561	89	2
Biomedical	19,448	3892	144,683	7.44	28	0.001870	20	2
GoogleNews	11,108	8110	69,229	6.23	14	0.000764	152	2
M10	8355	1696	49,385	5.91	21	0.003411	10	1
DBLP	54,595	1513	294,757	5.40	21	0.003527	4	1
PascalFlicker	4834	3431	25,980	5.37	19	0.001548	20	2
StackOverflow	16,407	2303	82,342	5.02	17	0.002145	20	2

**Table 3:** Topic Coherence on 20NewsGroups.

Model	Resources	TrainingCorpus	ExternalCorpus	glove.42B.300d		glove.6B.300d	
	Method	npmi	npmi	WETC${}_{c}$	WETC${}_{\mathrm{pw}}$	WETC${}_{c}$	WETC${}_{\mathrm{pw}}$
	PWE						
GSB	none	−0.16	−0.04	0.47	0.33	0.16	0.15
	pwe	−0.21	−0.05	0.50	0.32	0.16	0.15
	pwe/fine-tuning	−0.12	**−0.03**	0.48	0.34	**0.17**	0.17
GSM	none	−0.18	−0.05	0.50	0.32	0.15	0.15
	pwe	−0.21	−0.05	0.50	0.32	0.16	0.15
	pwe/fine-tuning	−0.21	−0.09	0.43	0.29	0.12	0.13
NSTM	none	−0.17	−0.05	0.53	0.33	0.13	0.16
	pwe	−0.19	−0.05	0.53	0.33	0.15	0.15
	pwe/fine-tuning	−0.16	−0.04	0.53	0.33	0.14	0.16
NVDM	none	−0.27	−0.05	0.47	0.30	0.14	0.14
	pwe	−0.22	−0.05	0.51	0.32	0.15	0.15
	pwe/fine-tuning	−0.27	−0.08	0.45	0.28	0.12	0.12
NVLDA	none	−0.18	−0.04	0.52	0.34	0.14	0.16
	pwe	−0.19	−0.04	0.52	0.33	0.14	0.15
	pwe/fine-tuning	**−0.10**	−0.04	**0.55**	**0.36**	0.16	**0.18**
ProdLDA	none	−0.21	−0.05	0.48	0.31	0.16	0.14
	pwe	−0.22	−0.05	0.50	0.32	0.15	0.15
	pwe/fine-tuning	−0.21	−0.09	0.46	0.29	0.12	0.12
RSB	none	−0.19	−0.06	0.48	0.31	0.15	0.14
	pwe	−0.18	−0.04	0.52	0.33	0.15	0.16
	pwe/fine-tuning	−0.26	−0.09	0.42	0.26	0.09	0.11
WLDA	none	−0.28	-	0.44	0.29	0.13	0.12
	pwe	−0.17	−0.04	0.52	0.33	0.15	0.16
	pwe/fine-tuning	−0.32	−0.07	0.51	0.32	0.15	0.14

**Table 4:** Topic Coherence on BBCNews.

Model	Resources	TrainingCorpus	ExternalCorpus	glove.42B.300d		glove.6B.300d	
	Method	npmi	npmi	WETC${}_{c}$	WETC${}_{\mathrm{pw}}$	WETC${}_{c}$	WETC${}_{\mathrm{pw}}$
	PWE						
GSB	none	−0.36	−0.09	0.44	0.28	0.13	0.13
	pwe	−0.42	−0.11	0.43	0.26	0.14	0.11
	pwe/fine-tuning	−0.35	−0.10	0.41	0.27	0.15	0.12
GSM	none	−0.40	−0.10	0.43	0.27	0.13	0.12
	pwe	−0.41	−0.10	0.44	0.27	0.14	0.12
	pwe/fine-tuning	−0.32	−0.09	0.45	0.29	**0.16**	0.14
NSTM	none	−0.40	−0.10	0.44	0.27	0.14	0.12
	pwe	−0.41	−0.10	0.46	0.27	0.13	0.12
	pwe/fine-tuning	−0.41	−0.09	0.45	0.27	0.14	0.12
NVDM	none	−0.44	−0.12	0.42	0.25	0.13	0.10
	pwe	−0.42	−0.11	0.43	0.26	0.12	0.11
	pwe/fine-tuning	−0.45	−0.17	0.38	0.22	0.12	0.08
NVLDA	none	−0.38	−0.09	0.46	0.28	0.15	0.13
	pwe	−0.39	−0.09	0.45	0.27	0.13	0.12
	pwe/fine-tuning	**−0.18**	**−0.03**	**0.53**	**0.36**	**0.16**	**0.20**
ProdLDA	none	−0.43	−0.13	0.41	0.25	0.12	0.10
	pwe	−0.42	−0.10	0.43	0.26	0.13	0.11
	pwe/fine-tuning	−0.27	−0.06	0.46	0.31	0.15	0.16
RSB	none	−0.40	−0.09	0.44	0.27	0.13	0.12
	pwe	−0.40	−0.09	0.45	0.27	0.13	0.12
	pwe/fine-tuning	−0.43	−0.18	0.34	0.21	0.14	0.09
WLDA	none	−0.41	-	0.44	0.27	0.14	0.12
	pwe	−0.43	−0.11	0.43	0.26	0.13	0.11
	pwe/fine-tuning	−0.39	−0.10	0.43	0.27	0.15	0.12

**Table 5:** Topic Coherence on Biomedical.

Model	Resources	TrainingCorpus	ExternalCorpus	glove.42B.300d		glove.6B.300d	
	Method	npmi	npmi	WETC${}_{c}$	WETC${}_{\mathrm{pw}}$	WETC${}_{c}$	WETC${}_{\mathrm{pw}}$
	PWE						
GSB	none	−0.52	−0.13	0.19	0.11	0.09	0.06
	pwe	−0.46	−0.12	0.22	0.24	0.12	0.19
	pwe/fine-tuning	−0.51	−0.14	0.19	0.23	0.09	0.18
GSM	none	−0.52	−0.12	0.19	0.10	0.09	0.06
	pwe	−0.47	−0.12	0.21	0.24	0.11	0.19
	pwe/fine-tuning	−0.49	−0.13	0.19	0.23	0.10	0.19
NSTM	none	−0.52	−0.13	0.21	0.12	0.10	0.06
	pwe	−0.49	−0.13	0.21	0.12	0.10	0.06
	pwe/fine-tuning	−0.52	−0.13	0.21	0.13	0.10	0.06
NVDM	none	−0.53	−0.12	0.18	0.09	0.08	0.05
	pwe	−0.51	−0.13	0.20	0.12	0.09	0.06
	pwe/fine-tuning	−0.52	−0.14	0.24	0.13	0.10	0.06
NVLDA	none	−0.47	−0.13	0.25	0.16	0.11	0.08
	pwe	−0.49	−0.13	0.21	0.23	0.10	0.18
	pwe/fine-tuning	**−0.15**	**−0.01**	**0.29**	**0.36**	**0.33**	**0.27**
ProdLDA	none	−0.50	−0.11	0.16	0.09	0.09	0.06
	pwe	−0.51	−0.13	0.21	0.23	0.09	0.18
	pwe/fine-tuning	−0.44	−0.12	0.25	0.25	0.14	0.19
RSB	none	−0.49	−0.14	0.19	0.12	0.10	0.07
	pwe	−0.48	−0.13	0.22	0.24	0.11	0.18
	pwe/fine-tuning	−0.47	−0.13	0.18	0.25	0.11	0.20
WLDA	none	−0.51	-	0.25	0.14	0.10	0.07
	pwe	−0.48	−0.13	0.20	0.13	0.11	0.07
	pwe/fine-tuning	−0.55	−0.11	0.15	0.08	0.09	0.06

**Table 6:** Topic Coherence on DBLP.

Model	Resources	TrainingCorpus	ExternalCorpus	glove.42B.300d		glove.6B.300d	
	Method	npmi	npmi	WETC${}_{c}$	WETC${}_{\mathrm{pw}}$	WETC${}_{c}$	WETC${}_{\mathrm{pw}}$
	PWE						
GSB	none	−0.52	−0.14	0.34	0.21	0.11	0.09
	pwe	−0.35	−0.10	0.37	0.25	0.13	0.11
	pwe/fine-tuning	−0.42	−0.13	0.35	0.23	0.12	0.10
GSM	none	−0.52	−0.14	0.33	0.21	0.12	0.09
	pwe	−0.38	−0.11	0.38	0.24	0.13	0.11
	pwe/fine-tuning	−0.43	−0.13	0.36	0.22	0.12	0.10
NSTM	none	−0.33	−0.08	0.37	0.26	0.13	0.13
	pwe	−0.42	−0.13	0.37	0.23	0.11	0.10
	pwe/fine-tuning	−0.31	−0.07	**0.40**	**0.27**	**0.15**	**0.14**
NVDM	none	−0.48	−0.14	0.34	0.22	0.11	0.09
	pwe	−0.47	−0.13	0.34	0.22	0.11	0.09
	pwe/fine-tuning	−0.45	−0.13	0.35	0.23	0.13	0.10
NVLDA	none	−0.41	−0.11	0.37	0.24	0.13	0.11
	pwe	−0.41	−0.12	0.37	0.24	0.13	0.10
	pwe/fine-tuning	**−0.29**	−0.09	0.39	**0.27**	**0.15**	0.13
ProdLDA	none	−0.43	−0.14	0.34	0.21	0.12	0.09
	pwe	−0.41	−0.12	0.37	0.24	0.13	0.11
	pwe/fine-tuning	−0.45	−0.14	0.35	0.21	0.12	0.09
RSB	none	−0.41	−0.13	0.36	0.23	0.13	0.10
	pwe	−0.37	−0.11	0.38	0.24	0.12	0.11
	pwe/fine-tuning	−0.37	−0.13	0.33	0.22	0.13	0.10
WLDA	none	−0.43	-	0.37	0.24	0.13	0.11
	pwe	−0.39	−0.11	0.38	0.25	0.13	0.11
	pwe/fine-tuning	−0.35	**−0.06**	0.38	0.26	0.13	0.12

**Table 7:** Topic Coherence on GoogleNews.

Model	Resources	TrainingCorpus	ExternalCorpus	glove.42B.300d		glove.6B.300d	
	Method	npmi	npmi	WETC${}_{c}$	WETC${}_{\mathrm{pw}}$	WETC${}_{c}$	WETC${}_{\mathrm{pw}}$
	PWE						
GSB	none	−0.52	−0.15	0.30	0.14	0.09	0.03
	pwe	**−0.48**	−0.16	0.27	**0.29**	0.09	**0.25**
	pwe/fine-tuning	−0.51	−0.17	0.27	0.22	0.08	0.15
GSM	none	−0.53	−0.16	0.28	0.13	0.09	0.03
	pwe	**−0.48**	−0.16	0.28	0.27	0.09	0.21
	pwe/fine-tuning	−0.51	−0.15	0.28	0.24	0.08	0.18
NSTM	none	−0.54	−0.16	0.32	0.17	0.10	0.05
	pwe	**−0.48**	−0.16	0.29	0.13	0.08	0.03
	pwe/fine-tuning	−0.53	−0.16	0.31	0.16	0.09	0.04
NVDM	none	−0.53	−0.15	0.25	0.11	0.07	0.02
	pwe	−0.50	−0.16	0.28	0.13	0.08	0.03
	pwe/fine-tuning	−0.51	−0.15	0.29	0.15	0.08	0.04
NVLDA	none	−0.50	−0.15	0.34	0.19	0.10	0.06
	pwe	−0.49	−0.16	0.28	0.24	0.08	0.17
	pwe/fine-tuning	−0.55	−0.14	0.39	0.27	0.12	0.19
ProdLDA	none	−0.51	−0.15	0.28	0.12	0.08	0.03
	pwe	−0.49	−0.16	0.27	0.22	0.08	0.15
	pwe/fine-tuning	−0.53	−0.18	0.29	0.22	0.09	0.14
RSB	none	−0.53	−0.18	0.30	0.15	0.09	0.04
	pwe	**−0.48**	−0.16	0.29	0.25	0.09	0.18
	pwe/fine-tuning	−0.58	−0.18	0.30	0.24	0.10	0.17
WLDA	none	−0.53	-	0.25	0.11	0.08	0.02
	pwe	−0.50	−0.16	0.28	0.13	0.08	0.03
	pwe/fine-tuning	−0.55	**−0.12**	**0.41**	0.24	**0.13**	0.10

**Table 8:** Topic Coherence on M10.

Model	Resources	TrainingCorpus	ExternalCorpus	glove.42B.300d		glove.6B.300d	
	Method	npmi	npmi	WETC${}_{c}$	WETC${}_{\mathrm{pw}}$	WETC${}_{c}$	WETC${}_{\mathrm{pw}}$
	PWE						
GSB	none	−0.54	−0.13	0.37	0.23	0.11	0.10
	pwe	−0.51	−0.11	0.41	0.25	0.13	0.11
	pwe/fine-tuning	−0.54	−0.13	0.38	0.22	0.11	0.10
GSM	none	−0.55	−0.14	0.37	0.22	0.11	0.10
	pwe	−0.51	−0.12	0.39	0.24	0.13	0.11
	pwe/fine-tuning	−0.54	−0.15	0.35	0.20	0.10	0.08
NSTM	none	−0.43	−0.08	0.42	0.27	0.13	0.13
	pwe	−0.52	−0.12	0.42	0.24	0.13	0.11
	pwe/fine-tuning	−0.45	−0.08	0.42	0.27	0.13	0.13
NVDM	none	−0.55	−0.14	0.36	0.22	0.12	0.09
	pwe	−0.54	−0.12	0.37	0.23	0.12	0.10
	pwe/fine-tuning	−0.53	−0.13	0.38	0.24	0.12	0.11
NVLDA	none	−0.51	−0.11	0.39	0.24	0.13	0.11
	pwe	−0.51	−0.12	0.39	0.24	0.12	0.11
	pwe/fine-tuning	**−0.41**	**−0.03**	**0.45**	**0.31**	**0.17**	**0.16**
ProdLDA	none	−0.52	−0.14	0.37	0.22	0.12	0.09
	pwe	−0.53	−0.12	0.38	0.24	0.12	0.10
	pwe/fine-tuning	−0.52	−0.13	0.35	0.22	0.11	0.10
RSB	none	−0.52	−0.13	0.37	0.23	0.11	0.10
	pwe	−0.50	−0.10	0.40	0.25	0.14	0.12
	pwe/fine-tuning	−0.53	−0.14	0.36	0.22	0.12	0.10
WLDA	none	−0.55	-	0.36	0.21	0.11	0.09
	pwe	−0.55	−0.13	0.38	0.23	0.12	0.10
	pwe/fine-tuning	−0.54	−0.11	0.40	0.25	0.13	0.11

**Table 9:** Topic Coherence on PascalFlicker.

Model	Resources	TrainingCorpus	ExternalCorpus	glove.42B.300d		glove.6B.300d	
	Method	npmi	npmi	WETC${}_{c}$	WETC${}_{\mathrm{pw}}$	WETC${}_{c}$	WETC${}_{\mathrm{pw}}$
	PWE						
GSB	none	−0.51	−0.10	0.26	0.16	0.08	0.06
	pwe	−0.46	−0.10	0.27	0.24	0.08	0.17
	pwe/fine-tuning	−0.49	−0.09	0.27	0.23	0.10	0.15
GSM	none	−0.52	−0.10	0.26	0.16	0.08	0.06
	pwe	−0.46	−0.10	0.26	0.24	0.09	0.17
	pwe/fine-tuning	−0.47	−0.09	0.26	0.21	0.09	0.14
NSTM	none	−0.52	−0.09	0.24	0.15	0.08	0.05
	pwe	−0.48	−0.09	0.24	0.15	0.09	0.05
	pwe/fine-tuning	−0.51	−0.09	0.25	0.15	0.08	0.05
NVDM	none	−0.52	−0.09	0.25	0.14	0.09	0.05
	pwe	−0.51	−0.09	0.25	0.14	0.08	0.05
	pwe/fine-tuning	−0.50	−0.10	0.27	0.15	0.08	0.06
NVLDA	none	−0.48	−0.10	0.28	0.18	0.09	0.07
	pwe	−0.45	−0.10	0.27	0.23	0.09	0.17
	pwe/fine-tuning	**−0.40**	**−0.07**	**0.45**	**0.34**	**0.16**	**0.25**
ProdLDA	none	−0.48	−0.09	0.25	0.14	0.08	0.05
	pwe	−0.49	−0.10	0.26	0.22	0.08	0.15
	pwe/fine-tuning	**−0.40**	**−0.07**	0.39	0.30	0.12	0.21
RSB	none	−0.48	−0.09	0.31	0.20	0.09	0.08
	pwe	−0.45	−0.10	0.26	0.24	0.08	0.18
	pwe/fine-tuning	−0.48	−0.10	0.32	0.27	0.11	0.19
WLDA	none	−0.49	-	0.26	0.15	0.08	0.05
	pwe	−0.52	−0.09	0.26	0.14	0.08	0.05
	pwe/fine-tuning	−0.54	−0.11	0.30	0.19	0.10	0.08

**Table 10:** Topic Coherence on SearchSnippets.

Model	Resources	TrainingCorpus	ExternalCorpus	glove.42B.300d		glove.6B.300d	
	Method	npmi	npmi	WETC${}_{c}$	WETC${}_{\mathrm{pw}}$	WETC${}_{c}$	WETC${}_{\mathrm{pw}}$
	PWE						
GSB	none	−0.49	−0.15	0.27	0.14	0.09	0.04
	pwe	−0.46	−0.14	0.32	0.28	0.12	0.20
	pwe/fine-tuning	−0.49	−0.17	0.26	0.22	0.10	0.14
GSM	none	−0.50	−0.16	0.25	0.13	0.09	0.03
	pwe	−0.46	−0.14	0.31	0.27	0.12	0.18
	pwe/fine-tuning	−0.48	−0.16	0.27	0.21	0.10	0.13
NSTM	none	−0.49	−0.13	0.32	0.19	0.11	0.06
	pwe	−0.47	−0.14	0.30	0.18	0.11	0.06
	pwe/fine-tuning	−0.49	−0.13	0.31	0.18	0.10	0.06
NVDM	none	−0.50	−0.15	0.26	0.13	0.09	0.03
	pwe	−0.48	−0.14	0.31	0.17	0.10	0.05
	pwe/fine-tuning	−0.48	−0.13	0.32	0.20	0.11	0.06
NVLDA	none	−0.46	−0.12	0.36	0.22	0.13	0.09
	pwe	−0.45	−0.13	0.30	0.26	0.11	0.17
	pwe/fine-tuning	**−0.26**	**−0.06**	**0.48**	**0.36**	**0.18**	**0.24**
ProdLDA	none	−0.48	−0.15	0.28	0.16	0.11	0.05
	pwe	−0.48	−0.14	0.31	0.24	0.11	0.14
	pwe/fine-tuning	−0.42	−0.14	0.30	0.24	0.14	0.15
RSB	none	−0.47	−0.15	0.27	0.15	0.10	0.05
	pwe	−0.46	−0.13	0.31	0.28	0.12	0.19
	pwe/fine-tuning	−0.48	−0.14	0.24	0.20	0.09	0.13
WLDA	none	−0.49	-	0.29	0.17	0.11	0.05
	pwe	−0.47	−0.13	0.32	0.18	0.11	0.06
	pwe/fine-tuning	−0.31	−0.07	0.44	0.33	0.17	0.17

**Table 11:** Topic Coherence on StackOverflow.

Model	Resources	TrainingCorpus	ExternalCorpus	glove.42B.300d		glove.6B.300d	
	Method	npmi	npmi	WETC${}_{c}$	WETC${}_{\mathrm{pw}}$	WETC${}_{c}$	WETC${}_{\mathrm{pw}}$
	PWE						
GSB	none	−0.53	−0.10	0.31	0.22	0.15	0.08
	pwe	−0.48	−0.10	0.30	0.31	0.17	0.21
	pwe/fine-tuning	−0.50	−0.12	0.27	0.29	0.14	0.17
GSM	none	−0.53	−0.10	0.30	0.21	0.14	0.08
	pwe	−0.48	−0.11	0.30	0.31	0.16	0.21
	pwe/fine-tuning	−0.49	−0.10	0.24	0.26	0.14	0.15
NSTM	none	−0.48	−0.10	0.28	0.23	0.16	0.09
	pwe	−0.50	−0.10	0.31	0.23	0.14	0.09
	pwe/fine-tuning	−0.47	−0.10	0.30	0.24	0.15	0.09
NVDM	none	−0.55	−0.10	0.25	0.17	0.12	0.07
	pwe	−0.52	−0.10	0.30	0.22	0.14	0.08
	pwe/fine-tuning	−0.53	−0.09	0.30	0.22	0.14	0.08
NVLDA	none	−0.48	−0.09	0.32	0.25	0.16	0.10
	pwe	−0.50	−0.10	0.29	0.30	0.15	0.20
	pwe/fine-tuning	**−0.26**	**−0.05**	**0.37**	**0.39**	**0.25**	**0.24**
ProdLDA	none	−0.51	−0.10	0.25	0.19	0.12	0.07
	pwe	−0.52	−0.10	0.29	0.28	0.15	0.17
	pwe/fine-tuning	−0.44	−0.12	0.26	0.29	0.17	0.16
RSB	none	−0.49	−0.11	0.26	0.22	0.17	0.08
	pwe	−0.48	−0.09	0.30	0.31	0.15	0.21
	pwe/fine-tuning	−0.48	−0.13	0.19	0.28	0.17	0.16
WLDA	none	−0.53	-	0.31	0.23	0.15	0.09
	pwe	−0.53	−0.10	0.29	0.22	0.15	0.08
	pwe/fine-tuning	−0.51	−0.09	0.34	0.28	0.18	0.11

**Table 12:** Topic Coherence on TrecTweet.

Model	Resources	TrainingCorpus	ExternalCorpus	glove.42B.300d		glove.6B.300d	
	Method	npmi	npmi	WETC${}_{c}$	WETC${}_{\mathrm{pw}}$	WETC${}_{c}$	WETC${}_{\mathrm{pw}}$
	PWE						
GSB	none	−0.53	−0.14	0.29	0.14	0.08	0.04
	pwe	**−0.50**	−0.14	0.29	0.24	0.09	**0.17**
	pwe/fine-tuning	−0.54	−0.15	0.29	0.23	0.08	0.15
GSM	none	−0.53	−0.14	0.28	0.14	0.08	0.03
	pwe	**−0.50**	−0.14	0.27	0.24	0.08	**0.17**
	pwe/fine-tuning	−0.53	−0.14	0.28	0.22	0.09	0.14
NSTM	none	−0.53	−0.13	0.29	0.15	0.09	0.04
	pwe	−0.51	−0.14	0.29	0.14	0.08	0.03
	pwe/fine-tuning	−0.53	−0.14	0.29	0.15	0.09	0.04
NVDM	none	−0.51	−0.15	0.28	0.14	0.08	0.04
	pwe	−0.51	−0.14	0.28	0.14	0.08	0.03
	pwe/fine-tuning	−0.52	−0.14	0.29	0.15	0.09	0.04
NVLDA	none	−0.51	−0.14	0.32	0.18	0.09	0.05
	pwe	**−0.50**	−0.14	0.29	0.24	0.09	**0.17**
	pwe/fine-tuning	−0.55	**−0.12**	**0.35**	**0.27**	**0.11**	**0.17**
ProdLDA	none	−0.53	−0.14	0.28	0.13	0.08	0.03
	pwe	−0.51	−0.14	0.29	0.21	0.09	0.13
	pwe/fine-tuning	−0.55	−0.14	0.32	0.25	0.10	0.16
RSB	none	−0.53	−0.15	0.30	0.16	0.09	0.04
	pwe	**−0.50**	−0.13	0.30	0.25	0.09	**0.17**
	pwe/fine-tuning	−0.57	−0.15	0.28	**0.27**	0.10	**0.17**
WLDA	none	**−0.50**	-	0.29	0.14	0.09	0.04
	pwe	−0.51	−0.14	0.27	0.14	0.08	0.03
	pwe/fine-tuning	−0.54	−0.15	0.34	0.18	0.10	0.06

**Table 13:** Topic Diversity on 20NewsGroups.

Model	PWE	Topic Diversity	Inverted RBO	MSCD	TCD (42B)	TCD (6B)
GSB	none	0.78	0.99	0.08	0.17	0.60
	pwe	0.79	0.99	0.20	−0.18	0.67
	pwe/fine-tuning	0.68	0.98	0.31	0.15	0.52
GSM	none	0.84	**1.00**	0.09	0.17	0.66
	pwe	0.85	**1.00**	0.21	0.18	0.71
	pwe/fine-tuning	0.72	0.99	0.39	0.19	0.77
NSTM	none	0.83	0.99	0.93	0.17	0.74
	pwe	0.85	**1.00**	0.91	0.17	0.68
	pwe/fine-tuning	0.82	0.99	0.91	0.17	0.75
NVDM	none	0.70	0.99	0.18	0.19	0.73
	pwe	0.85	**1.00**	0.21	0.18	0.71
	pwe/fine-tuning	0.83	**1.00**	0.55	0.20	0.84
NVLDA	none	**0.91**	**1.00**	0.07	0.17	0.70
	pwe	0.82	0.99	0.19	0.17	0.83
	pwe/fine-tuning	0.64	0.99	0.35	0.15	0.65
ProdLDA	none	0.77	0.99	0.11	0.18	0.68
	pwe	0.84	**1.00**	0.22	0.18	0.73
	pwe/fine-tuning	0.64	0.99	0.39	0.20	0.84
RSB	none	0.88	**1.00**	0.07	0.18	0.65
	pwe	0.78	0.99	0.21	0.17	0.69
	pwe/fine-tuning	0.62	0.99	0.44	**0.21**	**0.94**
WLDA	none	0.78	0.99	0.55	0.20	0.77
	0pwe	0.83	**1.00**	0.26	0.17	0.72
	pwe/fine-tuning	0.56	0.98	**0.00**	0.18	0.73

**Table 14:** Topic Diversity on BBCNews.

Model	PWE	Topic Diversity	Inverted RBO	MSCD	TCD (42B)	TCD (6B)
GSB	none	0.85	0.99	0.07	0.21	0.80
	pwe	0.90	**1.00**	0.15	0.22	0.75
	pwe/fine-tuning	0.87	**1.00**	0.25	0.20	0.71
GSM	none	0.92	**1.00**	0.07	0.22	0.79
	pwe	0.91	**1.00**	0.15	0.22	0.80
	pwe/fine-tuning	0.84	**1.00**	0.30	0.19	0.68
NSTM	none	**1.00**	**1.00**	0.97	0.21	0.74
	pwe	0.90	**1.00**	0.86	0.22	0.80
	pwe/fine-tuning	0.95	**1.00**	0.76	0.21	0.74
NVDM	none	0.92	**1.00**	0.05	0.23	0.80
	pwe	0.92	**1.00**	0.18	0.23	0.78
	pwe/fine-tuning	0.88	**1.00**	0.65	**0.27**	0.81
NVLDA	none	0.97	**1.00**	0.06	0.21	0.74
	pwe	0.91	**1.00**	0.17	0.22	0.78
	pwe/fine-tuning	0.70	0.99	0.43	0.15	0.69
ProdLDA	none	0.81	0.99	0.05	0.24	**0.85**
	pwe	0.91	**1.00**	0.16	0.23	0.75
	pwe/fine-tuning	0.69	0.99	0.39	0.18	0.71
RSB	none	0.95	**1.00**	0.06	0.21	0.78
	pwe	0.88	**1.00**	0.19	0.21	0.80
	pwe/fine-tuning	0.73	0.99	0.40	**0.27**	0.72
WLDA	none	0.94	**1.00**	**0.04**	0.22	0.75
	pwe	0.90	**1.00**	0.18	0.22	0.79
	pwe/fine-tuning	0.85	**1.00**	0.65	0.21	0.71

**Table 15:** Topic Diversity on Biomedical.

Model	PWE	Topic Diversity	Inverted RBO	MSCD	TCD (42B)	TCD (6B)
GSB	none	0.96	**1.00**	0.08	0.56	0.93
	pwe	0.92	**1.00**	0.19	0.49	0.85
	pwe/fine-tuning	0.95	**1.00**	0.35	0.54	0.89
GSM	none	0.95	**1.00**	0.08	0.55	0.93
	pwe	0.93	**1.00**	0.17	0.50	0.86
	pwe/fine-tuning	0.91	**1.00**	0.30	0.53	0.89
NSTM	none	**1.00**	**1.00**	0.79	0.50	0.90
	pwe	0.95	**1.00**	0.84	0.49	0.86
	pwe/fine-tuning	**1.00**	**1.00**	0.80	0.53	0.91
NVDM	none	0.85	**1.00**	0.19	0.59	**0.97**
	pwe	0.93	**1.00**	0.11	0.51	0.94
	pwe/fine-tuning	0.95	**1.00**	0.17	0.46	0.90
NVLDA	none	0.97	**1.00**	0.06	0.45	0.85
	pwe	0.92	**1.00**	0.13	0.54	0.89
	pwe/fine-tuning	0.35	0.96	0.49	0.26	0.37
ProdLDA	none	0.78	0.99	0.12	0.67	0.92
	pwe	0.94	**1.00**	0.14	0.49	0.90
	pwe/fine-tuning	0.85	0.99	0.43	0.39	0.78
RSB	none	0.91	**1.00**	0.08	0.56	0.89
	pwe	0.88	**1.00**	0.19	0.49	0.85
	pwe/fine-tuning	0.62	0.98	0.44	0.62	0.87
WLDA	none	0.91	**1.00**	0.26	0.45	0.88
	pwe	0.92	**1.00**	0.22	0.52	0.87
	pwe/fine-tuning	0.73	0.99	**0.00**	**0.71**	**0.97**

**Table 16:** Topic Diversity on DBLP.

Model	PWE	Topic Diversity	Inverted RBO	MSCD	TCD (42B)	TCD (6B)
GSB	none	0.86	**1.00**	0.09	0.27	0.88
	pwe	0.83	**1.00**	0.29	0.24	0.81
	pwe/fine-tuning	0.89	**1.00**	0.27	0.27	0.83
GSM	none	0.86	**1.00**	0.09	0.28	0.86
	pwe	0.86	**1.00**	0.28	0.25	0.85
	pwe/fine-tuning	0.80	0.99	0.18	0.28	0.85
NSTM	none	0.71	0.98	0.76	0.27	**0.89**
	pwe	0.87	**1.00**	0.91	0.26	**0.89**
	pwe/fine-tuning	0.70	0.98	0.77	0.26	0.82
NVDM	none	0.88	**1.00**	0.58	0.27	0.87
	pwe	0.87	**1.00**	0.21	0.26	0.85
	pwe/fine-tuning	**0.90**	**1.00**	0.12	0.27	0.85
NVLDA	none	**0.90**	**1.00**	0.06	0.25	0.81
	pwe	0.87	**1.00**	0.29	0.25	0.80
	pwe/fine-tuning	0.74	0.99	0.11	0.22	0.72
ProdLDA	none	0.82	0.99	0.12	**0.31**	0.85
	pwe	0.87	**1.00**	0.26	0.25	0.79
	pwe/fine-tuning	0.82	**1.00**	0.10	0.29	0.85
RSB	none	0.86	**1.00**	0.18	0.27	0.81
	pwe	0.65	0.98	0.34	0.25	0.84
	pwe/fine-tuning	0.68	0.99	0.40	0.27	0.81
WLDA	none	0.85	**1.00**	0.38	0.25	0.79
	pwe	0.84	**1.00**	0.26	0.24	0.81
	pwe/fine-tuning	0.78	0.99	**0.00**	0.24	0.80

**Table 17:** Topic Diversity on GoogleNews.

Model	PWE	Topic Diversity	Inverted RBO	MSCD	TCD (42B)	TCD (6B)
GSB	none	0.98	**1.00**	0.09	0.38	0.96
	pwe	0.96	**1.00**	0.13	0.43	0.95
	pwe/fine-tuning	0.96	**1.00**	0.34	0.45	0.98
GSM	none	0.98	**1.00**	0.09	0.41	0.97
	pwe	0.97	**1.00**	0.12	0.42	0.97
	pwe/fine-tuning	0.91	**1.00**	0.43	0.41	0.96
NSTM	none	**1.00**	**1.00**	0.84	0.34	0.93
	pwe	0.99	**1.00**	0.69	0.40	0.98
	pwe/fine-tuning	**1.00**	**1.00**	0.81	0.36	0.94
NVDM	none	0.96	**1.00**	0.07	**0.50**	**0.99**
	pwe	0.97	**1.00**	**0.06**	0.43	**0.99**
	pwe/fine-tuning	0.96	**1.00**	0.10	0.39	0.95
NVLDA	none	0.99	**1.00**	**0.06**	0.32	0.90
	pwe	0.97	**1.00**	0.10	0.44	0.97
	pwe/fine-tuning	0.72	0.99	0.45	0.27	0.86
ProdLDA	none	0.90	**1.00**	0.11	0.43	0.97
	pwe	0.97	**1.00**	0.09	0.45	0.98
	pwe/fine-tuning	0.91	**1.00**	0.49	0.38	0.97
RSB	none	0.89	**1.00**	0.14	0.39	0.93
	pwe	0.87	**1.00**	0.20	0.43	0.96
	pwe/fine-tuning	0.71	0.99	0.40	0.37	0.92
WLDA	none	0.93	**1.00**	0.21	0.47	0.98
	pwe	0.92	**1.00**	0.11	0.41	0.97
	pwe/fine-tuning	0.82	0.99	0.24	0.25	0.82

**Table 18:** Topic Diversity on M10.

Model	PWE	Topic Diversity	Inverted RBO	MSCD	TCD (42B)	TCD (6B)
GSB	none	**0.91**	**1.00**	0.08	0.26	0.84
	pwe	0.86	**1.00**	0.22	0.23	0.81
	pwe/fine-tuning	0.89	**1.00**	0.28	0.25	0.88
GSM	none	0.89	**1.00**	0.08	0.27	0.85
	pwe	0.86	**1.00**	0.20	0.25	0.83
	pwe/fine-tuning	0.77	0.99	0.32	**0.29**	**0.90**
NSTM	none	0.75	0.98	0.77	0.23	0.87
	pwe	0.84	0.99	0.90	0.24	0.82
	pwe/fine-tuning	0.82	0.99	0.75	0.24	0.86
NVDM	none	0.85	**1.00**	**0.04**	0.28	0.86
	pwe	0.87	**1.00**	0.21	0.26	0.84
	pwe/fine-tuning	0.89	**1.00**	0.32	0.25	0.86
NVLDA	none	**0.91**	**1.00**	0.07	0.25	0.83
	pwe	0.87	**1.00**	0.22	0.24	0.83
	pwe/fine-tuning	0.64	0.99	0.20	0.19	0.68
ProdLDA	none	0.79	0.99	0.05	0.28	0.88
	pwe	0.87	**1.00**	0.19	0.26	0.84
	pwe/fine-tuning	0.81	0.99	0.20	0.27	0.87
RSB	none	0.87	**1.00**	0.15	0.25	0.83
	pwe	0.67	0.98	0.24	0.24	0.80
	pwe/fine-tuning	0.69	0.99	0.41	0.26	0.82
WLDA	none	0.87	**1.00**	0.16	**0.29**	**0.90**
	pwe	0.79	0.99	0.33	0.26	0.82
	pwe/fine-tuning	0.85	**1.00**	0.44	0.23	0.80

**Table 19:** Topic Diversity on PascalFlicker.

Model	PWE	Topic Diversity	Inverted RBO	MSCD	TCD (42B)	TCD (6B)
GSB	none	0.95	**1.00**	0.07	0.41	0.98
	pwe	0.91	**1.00**	0.17	0.40	0.97
	pwe/fine-tuning	0.93	**1.00**	0.55	0.38	0.95
GSM	none	0.94	**1.00**	0.07	0.42	0.97
	pwe	0.93	**1.00**	0.17	0.42	0.96
	pwe/fine-tuning	0.89	**1.00**	0.61	0.39	0.97
NSTM	none	**1.00**	**1.00**	0.79	**0.48**	**0.99**
	pwe	0.95	**1.00**	0.85	0.44	0.95
	pwe/fine-tuning	**1.00**	**1.00**	0.78	0.45	0.98
NVDM	none	0.93	**1.00**	0.05	0.46	0.98
	pwe	0.93	**1.00**	0.17	0.44	**0.99**
	pwe/fine-tuning	0.92	**1.00**	0.24	0.40	0.98
NVLDA	none	0.95	**1.00**	0.06	0.38	0.96
	pwe	0.91	**1.00**	0.18	0.40	0.97
	pwe/fine-tuning	0.49	0.98	0.75	0.18	0.71
ProdLDA	none	0.77	0.99	0.07	0.47	0.98
	pwe	0.93	**1.00**	0.16	0.43	0.98
	pwe/fine-tuning	0.59	0.97	0.82	0.22	0.84
RSB	none	0.91	**1.00**	0.09	0.32	0.94
	pwe	0.86	**1.00**	0.18	0.42	0.98
	pwe/fine-tuning	0.62	0.99	0.53	0.29	0.90
WLDA	none	0.94	**1.00**	**0.03**	0.43	0.98
	pwe	0.90	**1.00**	0.17	0.43	**0.99**
	pwe/fine-tuning	0.83	0.99	0.40	0.34	0.95

**Table 20:** Topic Diversity on SearchSnippets.

Model	PWE	Topic Diversity	Inverted RBO	MSCD	TCD (42B)	TCD (6B)
GSB	none	0.97	**1.00**	0.07	0.41	0.94
	pwe	0.94	**1.00**	0.20	0.32	0.81
	pwe/fine-tuning	0.96	**1.00**	0.34	0.42	0.91
GSM	none	0.95	**1.00**	0.07	0.45	0.93
	pwe	0.94	**1.00**	0.16	0.34	0.83
	pwe/fine-tuning	0.93	**1.00**	0.33	0.41	0.92
NSTM	none	**1.00**	**1.00**	0.86	0.32	0.87
	pwe	0.96	**1.00**	0.81	0.34	0.85
	pwe/fine-tuning	**1.00**	**1.00**	0.83	0.33	0.89
NVDM	none	0.90	**1.00**	0.07	0.45	**0.95**
	pwe	0.94	**1.00**	0.10	0.34	0.87
	pwe/fine-tuning	0.96	**1.00**	0.14	0.32	0.86
NVLDA	none	0.98	**1.00**	**0.06**	0.28	0.78
	pwe	0.95	**1.00**	0.13	0.33	0.85
	pwe/fine-tuning	0.51	0.97	0.64	0.17	0.68
ProdLDA	none	0.88	**1.00**	0.07	0.37	0.87
	pwe	0.95	**1.00**	0.12	0.34	0.89
	pwe/fine-tuning	0.78	0.99	0.56	0.32	0.82
RSB	none	0.92	**1.00**	0.08	0.43	0.91
	pwe	0.86	0.99	0.23	0.33	0.82
	pwe/fine-tuning	0.78	0.99	0.39	**0.48**	0.94
WLDA	none	0.92	**1.00**	0.25	0.36	0.89
	pwe	0.93	**1.00**	0.15	0.34	0.86
	pwe/fine-tuning	0.34	0.96	0.72	0.18	0.64

**Table 21:** Topic Diversity on StackOverflow.

Model	PWE	Topic Diversity	Inverted RBO	MSCD	TCD (42B)	TCD (6B)
GSB	none	0.94	**1.00**	0.09	0.29	0.70
	pwe	0.89	**1.00**	0.19	0.28	0.69
	pwe/fine-tuning	0.93	**1.00**	0.34	0.34	0.71
GSM	none	0.93	**1.00**	0.09	0.32	0.74
	pwe	0.90	**1.00**	0.20	0.34	0.68
	pwe/fine-tuning	0.79	0.99	0.38	0.37	0.69
NSTM	none	0.95	**1.00**	0.77	0.32	0.71
	pwe	0.90	**1.00**	0.89	0.28	0.74
	pwe/fine-tuning	**0.96**	**1.00**	0.78	0.32	0.71
NVDM	none	0.82	0.99	0.12	0.43	**0.82**
	pwe	0.89	**1.00**	0.18	0.34	0.72
	pwe/fine-tuning	0.92	**1.00**	0.25	0.32	0.75
NVLDA	none	0.94	**1.00**	**0.06**	0.29	0.69
	pwe	0.88	**1.00**	0.20	0.34	0.69
	pwe/fine-tuning	0.46	0.97	0.23	0.22	0.47
ProdLDA	none	0.75	0.99	**0.06**	0.38	0.79
	pwe	0.90	**1.00**	0.19	0.30	0.70
	pwe/fine-tuning	0.77	0.99	0.32	0.34	0.63
RSB	none	0.88	**1.00**	0.13	0.37	0.64
	pwe	0.71	0.99	0.25	0.33	0.68
	pwe/fine-tuning	0.65	0.99	0.39	**0.49**	0.70
WLDA	none	0.88	**1.00**	0.40	0.29	0.71
	pwe	0.84	**1.00**	0.30	0.34	0.71
	pwe/fine-tuning	0.83	**1.00**	0.16	0.24	0.59

**Table 22:** Topic Diversity on TrecTweet.

Model	PWE	Topic Diversity	Inverted RBO	MSCD	TCD (42B)	TCD (6B)
GSB	none	0.97	**1.00**	0.06	0.41	0.97
	pwe	0.95	**1.00**	0.15	0.42	0.96
	pwe/fine-tuning	0.94	**1.00**	0.43	0.36	0.96
GSM	none	0.97	**1.00**	0.06	0.42	0.96
	pwe	0.96	**1.00**	0.14	0.43	0.96
	pwe/fine-tuning	0.95	**1.00**	0.53	0.40	0.96
NSTM	none	**1.00**	**1.00**	0.55	0.42	0.96
	pwe	0.97	**1.00**	0.79	0.41	0.97
	pwe/fine-tuning	**1.00**	**1.00**	0.77	0.43	0.96
NVDM	none	0.96	**1.00**	**0.05**	0.41	0.96
	pwe	0.95	**1.00**	0.14	**0.44**	**0.98**
	pwe/fine-tuning	0.97	**1.00**	0.25	0.39	0.96
NVLDA	none	0.98	**1.00**	0.06	0.36	0.94
	pwe	0.95	**1.00**	0.14	0.40	0.96
	pwe/fine-tuning	0.83	0.99	0.41	0.26	0.87
ProdLDA	none	0.92	**1.00**	**0.05**	0.42	0.97
	pwe	0.96	**1.00**	0.14	0.43	0.96
	pwe/fine-tuning	0.76	0.99	0.64	0.31	0.92
RSB	none	0.92	**1.00**	0.08	0.36	0.95
	pwe	0.88	**1.00**	0.19	0.39	0.95
	pwe/fine-tuning	0.52	0.98	0.57	0.35	0.96
WLDA	none	0.96	**1.00**	**0.05**	0.40	0.96
	pwe	0.94	**1.00**	0.12	**0.44**	0.97
	pwe/fine-tuning	0.87	**1.00**	0.44	0.33	0.94

**Table 23:** Classification and Clustering performance on 20NewsGroups.

Model	Classifier	SVM (Linear)				SVM (rbm)				-	
	Method	ACC	Precision	Recall	F1	ACC	Precision	Recall	F1	NMI	Purity
	PWE										
GSB	none	0.34	0.32	0.32	0.29	0.30	0.29	0.28	0.27	0.18	0.14
	pwe	0.34	0.33	0.33	0.31	0.34	0.34	0.32	0.31	0.26	0.25
	pwe/fine-tuning	0.48	0.46	0.47	0.45	0.49	0.48	0.48	0.47	0.33	**0.33**
GSM	none	0.30	0.28	0.28	0.25	0.25	0.23	0.24	0.21	0.20	0.14
	pwe	0.31	0.28	0.30	0.27	0.30	0.29	0.28	0.27	0.24	0.21
	pwe/fine-tuning	0.45	0.42	0.44	0.41	0.45	0.43	0.43	0.42	**0.35**	**0.33**
NSTM	none	0.22	0.23	0.21	0.18	0.07	0.15	0.07	0.03	0.02	0.07
	pwe	0.14	0.18	0.13	0.11	0.08	0.29	0.08	0.06	0.04	0.08
	pwe/fine-tuning	0.19	0.19	0.18	0.15	0.07	0.05	0.07	0.02	0.01	0.06
NVDM	none	0.36	0.34	0.35	0.32	0.23	0.22	0.22	0.20	0.02	0.07
	pwe	0.38	0.36	0.37	0.34	0.33	0.33	0.32	0.32	0.11	0.13
	pwe/fine-tuning	**0.54**	**0.53**	**0.52**	**0.50**	**0.55**	**0.53**	**0.53**	**0.53**	0.21	0.20
NVLDA	none	0.31	0.30	0.30	0.27	0.26	0.29	0.25	0.25	0.06	0.11
	pwe	0.20	0.20	0.19	0.15	0.12	0.15	0.11	0.08	0.07	0.09
	pwe/fine-tuning	0.36	0.34	0.35	0.31	0.27	0.34	0.26	0.23	0.33	0.29
ProdLDA	none	0.18	0.15	0.17	0.12	0.11	0.08	0.10	0.06	0.05	0.07
	pwe	0.18	0.17	0.17	0.13	0.10	0.14	0.10	0.06	0.05	0.08
	pwe/fine-tuning	0.36	0.34	0.34	0.30	0.27	0.33	0.26	0.23	0.32	0.27
RSB	none	0.15	0.09	0.14	0.07	0.14	0.09	0.13	0.06	0.18	0.13
	pwe	0.11	0.05	0.10	0.05	0.10	0.03	0.09	0.03	0.08	0.09
	pwe/fine-tuning	0.30	0.26	0.29	0.24	0.30	0.29	0.29	0.25	0.21	0.15
WLDA	none	0.07	0.10	0.07	0.03	0.07	0.13	0.07	0.03	0.00	0.06
	pwe	0.07	0.11	0.07	0.04	0.07	0.12	0.07	0.04	0.00	0.06
	pwe/fine-tuning	0.07	0.10	0.07	0.04	0.07	0.10	0.07	0.04	0.00	0.06

**Table 24:** Classification and Clustering performance on BBCNews.

Model	Classifier	SVM (Linear)				SVM (rbm)				-	
	Method	ACC	Precision	Recall	F1	ACC	Precision	Recall	F1	NMI	Purity
	PWE										
GSB	none	0.94	0.94	0.94	0.94	**0.95**	**0.95**	0.94	0.94	0.49	0.79
	pwe	0.89	0.89	0.89	0.89	0.89	0.89	0.88	0.88	0.42	0.81
	pwe/fine-tuning	0.92	0.92	0.92	0.92	0.91	0.92	0.91	0.91	0.51	0.86
GSM	none	0.94	0.94	0.94	0.94	0.94	0.94	0.94	0.94	0.51	0.78
	pwe	0.89	0.89	0.88	0.88	0.88	0.88	0.88	0.88	0.43	0.81
	pwe/fine-tuning	0.94	0.94	0.93	0.93	0.92	0.92	0.92	0.92	0.56	0.91
NSTM	none	0.57	0.69	0.56	0.57	0.52	0.66	0.51	0.51	0.16	0.51
	pwe	0.63	0.69	0.62	0.63	0.51	0.65	0.50	0.50	0.17	0.46
	pwe/fine-tuning	0.60	0.63	0.58	0.58	0.52	0.60	0.50	0.49	0.16	0.42
NVDM	none	0.92	0.92	0.91	0.91	0.83	0.83	0.83	0.83	0.21	0.41
	pwe	0.92	0.93	0.92	0.92	0.88	0.88	0.88	0.88	0.32	0.57
	pwe/fine-tuning	**0.95**	**0.95**	**0.95**	**0.95**	**0.95**	**0.95**	**0.95**	**0.95**	0.44	0.78
NVLDA	none	0.81	0.81	0.80	0.80	0.83	0.83	0.82	0.82	0.14	0.47
	pwe	0.87	0.88	0.87	0.87	0.78	0.82	0.77	0.78	0.40	0.72
	pwe/fine-tuning	0.92	0.93	0.92	0.92	0.90	0.91	0.90	0.90	0.61	**0.92**
ProdLDA	none	0.92	0.93	0.92	0.92	0.88	0.88	0.87	0.87	0.44	0.62
	pwe	0.85	0.87	0.85	0.85	0.74	0.81	0.73	0.74	0.33	0.59
	pwe/fine-tuning	0.93	0.93	0.93	0.93	0.91	0.91	0.91	0.91	**0.64**	**0.92**
RSB	none	0.43	0.29	0.39	0.27	0.43	0.28	0.39	0.27	0.30	0.41
	pwe	0.66	0.66	0.65	0.64	0.66	0.66	0.65	0.64	0.22	0.46
	pwe/fine-tuning	0.93	0.93	0.93	0.93	0.93	0.93	0.93	0.93	0.42	0.58
WLDA	none	0.34	0.36	0.32	0.27	0.28	0.27	0.25	0.16	0.01	0.24
	pwe	0.30	0.29	0.28	0.25	0.29	0.27	0.26	0.21	0.00	0.23
	pwe/fine-tuning	0.31	0.33	0.29	0.26	0.29	0.31	0.27	0.22	0.00	0.23

**Table 25:** Classification and Clustering performance on Biomedical.

Model	Classifier	SVM (Linear)				SVM (rbm)				-	
	Method	ACC	Precision	Recall	F1	ACC	Precision	Recall	F1	NMI	Purity
	PWE										
GSB	none	0.39	0.39	0.39	0.35	0.49	0.51	0.49	0.49	0.11	0.19
	pwe	0.22	0.20	0.22	0.18	0.25	0.25	0.25	0.23	0.07	0.14
	pwe/fine-tuning	0.31	0.30	0.31	0.28	0.36	0.36	0.36	0.35	0.13	0.21
GSM	none	0.40	0.38	0.40	0.36	0.49	0.51	0.49	0.49	0.11	0.20
	pwe	0.25	0.23	0.25	0.22	0.26	0.27	0.26	0.25	0.10	0.18
	pwe/fine-tuning	0.32	0.30	0.32	0.28	0.35	0.35	0.35	0.34	**0.15**	**0.23**
NSTM	none	0.25	0.23	0.24	0.21	0.29	0.28	0.29	0.28	0.00	0.05
	pwe	0.28	0.26	0.28	0.24	0.32	0.32	0.32	0.31	0.01	0.06
	pwe/fine-tuning	0.23	0.22	0.23	0.19	0.28	0.27	0.27	0.26	0.00	0.05
NVDM	none	0.42	0.41	0.42	0.38	0.55	0.57	0.55	0.55	0.06	0.14
	pwe	0.38	0.36	0.38	0.34	0.51	0.52	0.51	0.51	0.05	0.12
	pwe/fine-tuning	**0.45**	**0.44**	**0.44**	**0.41**	**0.57**	**0.59**	**0.57**	**0.57**	0.05	0.12
NVLDA	none	0.28	0.25	0.28	0.23	0.26	0.25	0.26	0.24	0.06	0.15
	pwe	0.06	0.02	0.06	0.01	0.06	0.02	0.06	0.01	0.00	0.05
	pwe/fine-tuning	0.10	0.07	0.10	0.06	0.07	0.04	0.07	0.03	0.03	0.08
ProdLDA	none	0.14	0.13	0.14	0.10	0.09	0.10	0.09	0.06	0.05	0.10
	pwe	0.06	0.01	0.05	0.01	0.06	0.01	0.05	0.01	0.00	0.05
	pwe/fine-tuning	0.11	0.08	0.11	0.07	0.07	0.05	0.07	0.03	0.03	0.08
RSB	none	0.18	0.14	0.18	0.13	0.20	0.17	0.19	0.16	0.04	0.09
	pwe	0.08	0.03	0.08	0.03	0.08	0.03	0.08	0.03	0.01	0.06
	pwe/fine-tuning	0.16	0.13	0.16	0.13	0.17	0.15	0.17	0.14	0.04	0.10
WLDA	none	0.08	0.06	0.08	0.04	0.08	0.06	0.08	0.04	0.00	0.05
	pwe	0.08	0.05	0.08	0.04	0.08	0.05	0.08	0.04	0.00	0.05
	pwe/fine-tuning	0.07	0.05	0.07	0.03	0.08	0.06	0.07	0.04	0.00	0.05

**Table 26:** Classification and Clustering performance on DBLP.

Model	Classifier	SVM (Linear)				SVM (rbm)				-	
	Method	ACC	Precision	Recall	F1	ACC	Precision	Recall	F1	NMI	Purity
	PWE										
GSB	none	0.69	0.65	0.58	0.58	0.74	0.70	0.65	0.67	0.04	0.46
	pwe	0.62	0.60	0.52	0.53	0.65	0.63	0.55	0.57	0.09	0.55
	pwe/fine-tuning	**0.72**	0.68	**0.62**	0.63	0.75	0.71	0.66	0.68	0.11	0.56
GSM	none	0.68	0.64	0.57	0.57	0.74	0.70	0.65	0.67	0.04	0.44
	pwe	0.64	0.61	0.55	0.56	0.66	0.64	0.56	0.58	0.11	0.59
	pwe/fine-tuning	**0.72**	**0.69**	**0.62**	**0.64**	0.74	0.71	0.66	0.67	0.14	0.61
NSTM	none	0.48	0.46	0.36	0.35	0.52	0.51	0.41	0.41	0.01	0.39
	pwe	0.50	0.47	0.40	0.39	0.56	0.55	0.46	0.47	0.00	0.38
	pwe/fine-tuning	0.46	0.46	0.34	0.32	0.50	0.50	0.40	0.40	0.00	0.38
NVDM	none	0.71	0.67	0.61	0.62	**0.76**	**0.72**	**0.68**	**0.70**	0.04	0.46
	pwe	0.64	0.62	0.54	0.54	0.72	0.69	0.63	0.65	0.02	0.42
	pwe/fine-tuning	0.71	0.67	0.61	0.61	**0.76**	**0.72**	**0.68**	**0.70**	0.04	0.45
NVLDA	none	0.53	0.50	0.42	0.41	0.54	0.53	0.43	0.43	0.03	0.45
	pwe	0.38	0.15	0.25	0.15	0.38	0.13	0.25	0.15	0.00	0.38
	pwe/fine-tuning	0.57	0.54	0.45	0.44	0.54	0.52	0.42	0.40	0.15	0.55
ProdLDA	none	0.66	0.65	0.54	0.56	0.63	0.66	0.52	0.54	**0.20**	**0.64**
	pwe	0.38	0.13	0.25	0.14	0.38	0.11	0.25	0.14	0.00	0.38
	pwe/fine-tuning	0.57	0.48	0.44	0.42	0.54	0.47	0.41	0.38	0.14	0.53
RSB	none	0.71	0.67	0.61	0.61	0.75	0.71	0.66	0.68	0.04	0.43
	pwe	0.41	0.23	0.31	0.24	0.40	0.19	0.30	0.22	0.02	0.39
	pwe/fine-tuning	0.69	0.64	0.58	0.58	0.70	0.66	0.61	0.62	0.11	0.50
WLDA	none	0.39	0.23	0.26	0.18	0.39	0.24	0.26	0.18	0.00	0.38
	pwe	0.39	0.25	0.26	0.18	0.39	0.28	0.26	0.18	0.00	0.38
	pwe/fine-tuning	0.39	0.24	0.26	0.17	0.39	0.27	0.26	0.17	0.00	0.38

**Table 27:** Classification and Clustering performance on GoogleNews.

Model	Classifier	SVM (Linear)				SVM (rbm)				-	
	Method	ACC	Precision	Recall	F1	ACC	Precision	Recall	F1	NMI	Purity
	PWE										
GSB	none	0.85	0.81	0.78	0.78	0.85	0.82	0.74	0.77	0.46	0.25
	pwe	0.55	0.37	0.36	0.33	0.52	0.30	0.30	0.27	0.63	0.41
	pwe/fine-tuning	0.85	0.77	0.74	0.74	0.83	0.76	0.70	0.71	0.54	0.27
GSM	none	0.84	0.82	0.75	0.77	0.85	0.82	0.74	0.77	0.47	0.25
	pwe	0.53	0.30	0.31	0.27	0.51	0.24	0.27	0.23	**0.68**	**0.46**
	pwe/fine-tuning	0.80	0.65	0.60	0.60	0.82	0.68	0.64	0.65	0.67	0.40
NSTM	none	0.15	0.04	0.04	0.03	0.09	0.02	0.02	0.02	0.00	0.04
	pwe	0.52	0.46	0.40	0.41	0.49	0.44	0.32	0.35	0.01	0.04
	pwe/fine-tuning	0.15	0.04	0.05	0.03	0.11	0.03	0.03	0.02	0.01	0.04
NVDM	none	0.87	**0.85**	**0.83**	**0.83**	**0.89**	**0.87**	**0.82**	**0.84**	0.30	0.15
	pwe	0.85	0.83	0.81	0.81	0.88	**0.87**	0.80	0.83	0.32	0.17
	pwe/fine-tuning	**0.88**	0.83	**0.83**	0.82	**0.89**	**0.87**	0.81	0.83	0.27	0.13
NVLDA	none	0.56	0.45	0.41	0.40	0.48	0.37	0.30	0.30	0.33	0.19
	pwe	0.14	0.03	0.04	0.03	0.14	0.03	0.04	0.03	0.17	0.12
	pwe/fine-tuning	0.32	0.08	0.12	0.08	0.29	0.07	0.10	0.07	0.53	0.30
ProdLDA	none	0.41	0.12	0.17	0.12	0.38	0.12	0.15	0.11	0.61	0.36
	pwe	0.11	0.02	0.03	0.02	0.10	0.02	0.03	0.02	0.11	0.08
	pwe/fine-tuning	0.35	0.08	0.13	0.09	0.33	0.08	0.12	0.08	0.59	0.32
RSB	none	0.59	0.49	0.48	0.47	0.59	0.48	0.47	0.47	0.34	0.12
	pwe	0.07	0.00	0.01	0.00	0.07	0.00	0.01	0.00	0.06	0.05
	pwe/fine-tuning	0.65	0.43	0.44	0.41	0.64	0.39	0.41	0.38	0.37	0.14
WLDA	none	0.05	0.01	0.01	0.01	0.06	0.01	0.01	0.01	0.00	0.04
	pwe	0.06	0.01	0.02	0.01	0.06	0.01	0.02	0.01	0.00	0.04
	pwe/fine-tuning	0.06	0.01	0.02	0.01	0.06	0.01	0.02	0.01	0.00	0.04

**Table 28:** Classification and Clustering performance on M10.

Model	Classifier	SVM (Linear)				SVM (rbm)				-	
	Method	ACC	Precision	Recall	F1	ACC	Precision	Recall	F1	NMI	Purity
	PWE										
GSB	none	0.66	0.62	0.59	0.59	0.73	0.74	0.67	0.68	0.14	0.34
	pwe	0.52	0.46	0.46	0.45	0.53	0.50	0.47	0.47	0.22	0.46
	pwe/fine-tuning	0.66	0.62	0.59	0.59	0.68	0.67	0.63	0.63	0.26	0.50
GSM	none	0.66	0.62	0.59	0.59	0.72	0.74	0.67	0.68	0.14	0.34
	pwe	0.54	0.49	0.48	0.48	0.54	0.50	0.48	0.48	0.24	0.50
	pwe/fine-tuning	0.66	0.63	0.60	0.60	0.68	0.68	0.62	0.63	0.30	0.54
NSTM	none	0.40	0.36	0.35	0.35	0.50	0.45	0.45	0.45	0.04	0.17
	pwe	0.41	0.39	0.36	0.36	0.49	0.49	0.44	0.44	0.00	0.14
	pwe/fine-tuning	0.36	0.33	0.32	0.31	0.44	0.42	0.40	0.40	0.01	0.14
NVDM	none	0.62	0.60	0.56	0.55	0.72	0.74	0.66	0.67	0.08	0.26
	pwe	0.57	0.53	0.50	0.50	0.70	0.73	0.64	0.65	0.07	0.26
	pwe/fine-tuning	**0.69**	**0.67**	**0.64**	**0.64**	**0.76**	**0.77**	**0.71**	**0.73**	0.10	0.28
NVLDA	none	0.41	0.37	0.36	0.35	0.43	0.40	0.38	0.38	0.08	0.28
	pwe	0.19	0.15	0.15	0.10	0.20	0.18	0.16	0.11	0.08	0.20
	pwe/fine-tuning	0.52	0.53	0.45	0.45	0.50	0.57	0.43	0.44	0.33	0.51
ProdLDA	none	0.64	0.58	0.57	0.56	0.56	0.63	0.50	0.52	**0.34**	**0.59**
	pwe	0.16	0.11	0.12	0.06	0.18	0.16	0.14	0.08	0.05	0.18
	pwe/fine-tuning	0.54	0.57	0.48	0.48	0.52	0.58	0.45	0.46	0.33	0.54
RSB	none	0.64	0.59	0.57	0.57	0.69	0.70	0.63	0.64	0.11	0.26
	pwe	0.22	0.13	0.18	0.12	0.21	0.13	0.18	0.11	0.05	0.19
	pwe/fine-tuning	0.64	0.57	0.58	0.57	0.66	0.59	0.59	0.59	0.21	0.32
WLDA	none	0.16	0.11	0.13	0.09	0.16	0.11	0.13	0.09	0.00	0.13
	pwe	0.16	0.11	0.13	0.08	0.16	0.13	0.13	0.09	0.00	0.13
	pwe/fine-tuning	0.16	0.12	0.13	0.08	0.16	0.12	0.13	0.08	0.00	0.13

**Table 29:** Classification and Clustering performance on PascalFlicker.

Model	Classifier	SVM (Linear)				SVM (rbm)				-	
	Method	ACC	Precision	Recall	F1	ACC	Precision	Recall	F1	NMI	Purity
	PWE										
GSB	none	0.34	0.34	0.34	0.31	0.41	0.45	0.42	0.41	0.14	0.19
	pwe	0.22	0.21	0.22	0.19	0.23	0.22	0.23	0.21	0.14	0.18
	pwe/fine-tuning	0.30	0.28	0.30	0.27	0.32	0.33	0.32	0.31	0.18	**0.21**
GSM	none	0.34	0.32	0.34	0.31	0.40	0.44	0.40	0.40	0.14	0.20
	pwe	0.22	0.21	0.22	0.19	0.22	0.22	0.22	0.20	0.15	0.19
	pwe/fine-tuning	0.31	0.31	0.32	0.29	0.31	0.32	0.31	0.29	**0.19**	**0.21**
NSTM	none	0.19	0.23	0.19	0.16	0.11	0.12	0.11	0.09	0.00	0.05
	pwe	0.14	0.13	0.15	0.11	0.13	0.13	0.13	0.10	0.01	0.06
	pwe/fine-tuning	0.11	0.10	0.11	0.06	0.09	0.07	0.09	0.04	0.00	0.05
NVDM	none	0.34	0.32	0.34	0.31	0.46	0.49	0.46	0.46	0.06	0.12
	pwe	0.35	0.33	0.35	0.32	0.46	0.50	0.46	0.47	0.09	0.14
	pwe/fine-tuning	**0.40**	**0.38**	**0.40**	**0.37**	**0.50**	**0.53**	**0.50**	**0.50**	0.10	0.15
NVLDA	none	0.25	0.24	0.26	0.22	0.26	0.26	0.26	0.25	0.11	0.15
	pwe	0.06	0.02	0.06	0.02	0.06	0.04	0.06	0.02	0.00	0.06
	pwe/fine-tuning	0.08	0.05	0.08	0.04	0.08	0.05	0.08	0.04	0.07	0.10
ProdLDA	none	0.30	0.29	0.30	0.27	0.23	0.27	0.23	0.21	0.13	0.16
	pwe	0.05	0.01	0.05	0.01	0.05	0.01	0.05	0.01	0.00	0.05
	pwe/fine-tuning	0.09	0.06	0.09	0.05	0.09	0.06	0.08	0.05	0.08	0.11
RSB	none	0.23	0.20	0.23	0.19	0.26	0.25	0.26	0.25	0.07	0.11
	pwe	0.10	0.05	0.10	0.04	0.10	0.03	0.10	0.03	0.08	0.10
	pwe/fine-tuning	0.17	0.13	0.17	0.13	0.17	0.14	0.17	0.13	0.10	0.11
WLDA	none	0.07	0.06	0.07	0.04	0.07	0.05	0.07	0.04	0.00	0.05
	pwe	0.06	0.05	0.06	0.04	0.07	0.05	0.07	0.04	0.00	0.05
	pwe/fine-tuning	0.06	0.04	0.06	0.04	0.06	0.04	0.06	0.03	0.00	0.05

**Table 30:** Classification and Clustering performance on SearchSnippets.

Model	Classifier	SVM (Linear)				SVM (rbm)				-	
	Method	ACC	Precision	Recall	F1	ACC	Precision	Recall	F1	NMI	Purity
	PWE										
GSB	none	0.51	0.54	0.48	0.49	0.78	0.80	0.76	0.78	0.08	0.33
	pwe	0.41	0.39	0.34	0.34	0.48	0.49	0.41	0.42	0.09	0.35
	pwe/fine-tuning	0.60	0.59	0.53	**0.54**	0.71	0.73	0.67	0.68	0.16	0.41
GSM	none	0.50	0.55	0.45	0.47	0.78	0.81	0.75	0.77	0.08	0.33
	pwe	0.45	0.42	0.38	0.38	0.48	0.53	0.41	0.42	0.13	0.40
	pwe/fine-tuning	**0.61**	**0.61**	**0.54**	**0.54**	0.70	0.72	0.65	0.67	**0.18**	**0.45**
NSTM	none	0.25	0.32	0.16	0.11	0.27	0.41	0.19	0.16	0.01	0.22
	pwe	0.29	0.43	0.20	0.16	0.26	0.59	0.17	0.13	0.02	0.23
	pwe/fine-tuning	0.24	0.29	0.15	0.10	0.25	0.32	0.17	0.13	0.01	0.22
NVDM	none	0.44	0.49	0.41	0.43	0.80	0.83	0.77	0.79	0.03	0.25
	pwe	0.44	0.50	0.40	0.41	0.78	0.82	0.76	0.78	0.03	0.25
	pwe/fine-tuning	0.52	0.55	0.51	0.52	**0.83**	**0.85**	**0.81**	**0.82**	0.04	0.26
NVLDA	none	0.37	0.36	0.31	0.31	0.40	0.42	0.33	0.34	0.05	0.28
	pwe	0.27	0.23	0.18	0.14	0.24	0.20	0.15	0.10	0.03	0.24
	pwe/fine-tuning	0.27	0.19	0.19	0.14	0.25	0.12	0.16	0.11	0.03	0.26
ProdLDA	none	0.33	0.36	0.25	0.24	0.46	0.56	0.38	0.40	0.06	0.30
	pwe	0.23	0.11	0.14	0.08	0.22	0.11	0.13	0.06	0.01	0.22
	pwe/fine-tuning	0.27	0.17	0.18	0.13	0.25	0.14	0.16	0.11	0.03	0.25
RSB	none	0.30	0.22	0.21	0.18	0.42	0.38	0.35	0.33	0.02	0.23
	pwe	0.22	0.09	0.13	0.07	0.22	0.05	0.13	0.06	0.01	0.22
	pwe/fine-tuning	0.32	0.28	0.24	0.21	0.38	0.35	0.30	0.30	0.03	0.25
WLDA	none	0.22	0.16	0.14	0.08	0.22	0.16	0.14	0.08	0.00	0.22
	pwe	0.22	0.14	0.14	0.08	0.23	0.17	0.14	0.08	0.00	0.22
	pwe/fine-tuning	0.23	0.15	0.14	0.09	0.23	0.16	0.14	0.09	0.00	0.22

**Table 31:** Classification and Clustering performance on StackOverflow.

Model	Classifier	SVM (Linear)				SVM (rbm)				-	
	Method	ACC	Precision	Recall	F1	ACC	Precision	Recall	F1	NMI	Purity
	PWE										
GSB	none	0.71	0.68	0.70	0.68	0.72	0.72	0.71	0.71	0.16	0.23
	pwe	0.70	0.71	0.70	0.70	0.72	0.78	0.72	0.74	0.50	0.60
	pwe/fine-tuning	0.68	0.66	0.67	0.66	0.69	0.69	0.68	0.68	0.35	0.44
GSM	none	0.71	0.69	0.70	0.69	0.72	0.71	0.71	0.71	0.17	0.24
	pwe	0.73	0.76	0.73	0.73	0.74	**0.81**	0.73	0.76	**0.59**	**0.70**
	pwe/fine-tuning	0.71	0.70	0.71	0.70	0.71	0.72	0.71	0.71	0.47	0.59
NSTM	none	0.22	0.20	0.21	0.19	0.25	0.24	0.24	0.24	0.00	0.06
	pwe	0.45	0.42	0.44	0.41	0.47	0.45	0.46	0.45	0.00	0.06
	pwe/fine-tuning	0.21	0.19	0.20	0.18	0.22	0.22	0.22	0.22	0.00	0.06
NVDM	none	0.74	0.72	0.74	0.72	0.76	0.76	0.75	0.76	0.13	0.19
	pwe	0.71	0.68	0.70	0.68	0.75	0.75	0.74	0.75	0.10	0.19
	pwe/fine-tuning	**0.78**	**0.77**	**0.77**	**0.77**	**0.79**	0.80	**0.79**	**0.79**	0.15	0.22
NVLDA	none	0.50	0.47	0.49	0.46	0.48	0.47	0.47	0.46	0.19	0.30
	pwe	0.07	0.02	0.06	0.02	0.07	0.01	0.06	0.02	0.00	0.06
	pwe/fine-tuning	0.22	0.15	0.21	0.15	0.15	0.12	0.14	0.10	0.15	0.16
ProdLDA	none	0.44	0.43	0.43	0.39	0.32	0.38	0.31	0.30	0.35	0.34
	pwe	0.06	0.01	0.06	0.01	0.06	0.02	0.06	0.01	0.00	0.06
	pwe/fine-tuning	0.29	0.25	0.28	0.23	0.18	0.17	0.17	0.14	0.21	0.19
RSB	none	0.64	0.61	0.63	0.60	0.66	0.64	0.65	0.64	0.18	0.17
	pwe	0.10	0.04	0.10	0.04	0.10	0.04	0.09	0.03	0.05	0.08
	pwe/fine-tuning	0.66	0.63	0.65	0.63	0.67	0.66	0.66	0.65	0.29	0.22
WLDA	none	0.09	0.07	0.09	0.05	0.09	0.08	0.09	0.05	0.00	0.06
	pwe	0.10	0.08	0.10	0.06	0.10	0.08	0.10	0.06	0.00	0.06
	pwe/fine-tuning	0.11	0.08	0.10	0.07	0.10	0.09	0.10	0.07	0.00	0.06

**Table 32:** Classification and Clustering performance on TrecTweet.

Model	Classifier	SVM (Linear)				SVM (rbm)				-	
	Method	ACC	Precision	Recall	F1	ACC	Precision	Recall	F1	NMI	Purity
	PWE										
GSB	none	-	-	-	-	-	-	-	-	0.50	0.39
	pwe	-	-	-	-	-	-	-	-	0.62	0.54
	pwe/fine-tuning	-	-	-	-	-	-	-	-	0.66	0.49
GSM	none	-	-	-	-	-	-	-	-	0.52	0.40
	pwe	-	-	-	-	-	-	-	-	0.65	0.58
	pwe/fine-tuning	-	-	-	-	-	-	-	-	**0.74**	0.56
NSTM	none	-	-	-	-	-	-	-	-	0.04	0.12
	pwe	-	-	-	-	-	-	-	-	0.03	0.13
	pwe/fine-tuning	-	-	-	-	-	-	-	-	0.00	0.10
NVDM	none	-	-	-	-	-	-	-	-	0.39	0.30
	pwe	-	-	-	-	-	-	-	-	0.37	0.27
	pwe/fine-tuning	-	-	-	-	-	-	-	-	0.43	0.33
NVLDA	none	-	-	-	-	-	-	-	-	0.44	0.36
	pwe	-	-	-	-	-	-	-	-	0.39	0.34
	pwe/fine-tuning	-	-	-	-	-	-	-	-	0.70	**0.59**
ProdLDA	none	-	-	-	-	-	-	-	-	0.60	0.50
	pwe	-	-	-	-	-	-	-	-	0.16	0.18
	pwe/fine-tuning	-	-	-	-	-	-	-	-	0.62	0.49
RSB	none	-	-	-	-	-	-	-	-	0.38	0.26
	pwe	-	-	-	-	-	-	-	-	0.17	0.18
	pwe/fine-tuning	-	-	-	-	-	-	-	-	0.41	0.28
WLDA	none	-	-	-	-	-	-	-	-	0.00	0.10
	pwe	-	-	-	-	-	-	-	-	0.01	0.10
	pwe/fine-tuning	-	-	-	-	-	-	-	-	0.00	0.10
